# Variation in the *SERPINA6/SERPINA1* locus alters morning plasma cortisol, hepatic corticosteroid binding globulin expression, gene expression in peripheral tissues, and risk of cardiovascular disease

**DOI:** 10.1038/s10038-020-00895-6

**Published:** 2021-01-20

**Authors:** Andrew A. Crawford, Sean Bankier, Elisabeth Altmaier, Catriona L. K. Barnes, David W. Clark, Raili Ermel, Nele Friedrich, Pim van der Harst, Peter K. Joshi, Ville Karhunen, Jari Lahti, Anubha Mahajan, Massimo Mangino, Maria Nethander, Alexander Neumann, Maik Pietzner, Katyayani Sukhavasi, Carol A. Wang, Stephan J. L. Bakker, Johan L. M. Bjorkegren, Harry Campbell, Johan Eriksson, Christian Gieger, Caroline Hayward, Marjo-Riitta Jarvelin, Stela McLachlan, Andrew P. Morris, Claes Ohlsson, Craig E. Pennell, Jackie Price, Igor Rudan, Arno Ruusalepp, Tim Spector, Henning Tiemeier, Henry Völzke, James F. Wilson, Tom Michoel, Nicolas J. Timpson, George Davey Smith, Brian R. Walker, on behalf of the CORtisol NETwork (CORNET) consortium, Dan Mellström

**Affiliations:** 1 BHF Centre for Cardiovascular Science, Queen’s Medical Research Institute, University of Edinburgh, Edinburgh, UK; 2 MRC Integrative Epidemiology Unit, University of Bristol, Bristol, UK; 3 Population Health Sciences, Bristol Medical School, University of Bristol, Bristol, UK; 4 Division of Genetics and Genomics, The Roslin Institute, The University of Edinburgh, Easter Bush, Midlothian EH25 9RG UK; 5 Research Unit of Molecular Epidemiology, Helmholtz Zentrum München-German Research Center for Environmental Health, Neuherberg, Germany; 6 Centre for Global Health Research, Usher Institute, University of Edinburgh, Teviot Place, Edinburgh EH8 9AG Scotland; 7 Department of Cardiac Surgery, Tartu University Hospital, Tartu, Estonia; 8 Institute of Clinical Chemistry and Laboratory Medicine, University Medicine Greifswald, 17475 Greifswald, Germany; 9 German Center for Cardiovascular Disease (DZHK e.V.), partner site Greifswald, 17475 Greifswald, Germany; 10 Division of Heart and Lungs, Department of Cardiology, University Medical Center Utrecht, Utrecht University, Utrecht, The Netherlands; 11 Department of Cardiology, University Medical Center Groningen, University of Groningen, Groningen, PO box 30.001, 9700 RB The Netherlands; 12 Department of Epidemiology and Biostatistics, Medical Research Council–Public Health England Centre for Environment and Health, Imperial College London, London, UK; 13 Centre for Life Course Health Research, Faculty of Medicine, University of Oulu, Oulu, Finland; 14 Department of Psychology and Logopedics, University of Helsinki, Helsinki, Finland; 15 Turku Institute of Advanced Studies, University of Turku, Turku, Finland; 16 Oxford Centre for Diabetes, Endocrinology and Metabolism, University of Oxford, Oxford, UK; 17 Wellcome Centre for Human Genetics, University of Oxford, Oxford, UK; 18 Department of Twin Research and Genetic Epidemiology, King’s College, Lambeth Palace Road, London, SE1 7EH UK; 19 NIHR Biomedical Research Centre at Guy’s and St Thomas’ Foundation Trust, London, UK; 20 Centre for Bone and Arthritis Research, Department of Internal Medicine and Clinical Nutrition, Institute of Medicine, The Sahlgrenska Academy, University of Gothenburg, Gothenburg, Sweden; 21 Bioinformatics Core Facility, Sahlgrenska Academy, University of Gothenburg, Gothenburg, Sweden; 22 Department of Child and Adolescent Psychiatry/Psychology, Erasmus University Medical Center Rotterdam, Rotterdam, The Netherlands; 23 Lady Davis Institute for Medical Research, Jewish General Hospital, Montreal, QC Canada; 24 School of Medicine and Public Health, Faculty of Medicine and Health, University of Newcastle, Newcastle, NSW 2308 Australia; 25 Department of Internal Medicine, University Medical Center Groningen, University of Groningen, Groningen, The Netherlands; 26 Integrated Cardio Metabolic Centre, Department of Medicine, Karolinska Institutet, Karolinska Universitetssjukhuset, Huddinge, Sweden; 27 Department of Genetics & Genomic Sciences, Institute of Genomics and Multiscale Biology, Icahn School of Medicine at Mount Sinai, New York, NY USA; 28 Clinical Gene Networks AB, Stockholm, Sweden; 29 Folkhälsan Research Center, Helsinki, Finland; 30 Department of General Practice and Primary Health Care, University of Helsinki and Helsinki University Hospital, Helsinki, Finland; 31 Department of Obstetrics & Gynaecology, Yong Loo Lin School of Medicine, National University Health System, National University of Singapore, Helsinki, Singapore; 32 Research Unit of Molecular Epidemiology, Helmholtz Zentrum München, German Research Center for Environmental Health, Neuherberg, Germany; 33 Institute of Epidemiology, Helmholtz Zentrum München, German Research Center for Environmental Health, Neuherberg, Germany; 34 German Center for Diabetes Research (DZD), Neuherberg, Germany; 35 MRC Human Genetics Unit, Institute of Genetics and Molecular Medicine, Western General Hospital University of Edinburgh, Edinburgh, EH4 2XU Scotland; 36 Centre for Life Course Health Research, Faculty of Medicine, University of Oulu, Oulu, Finland; 37 Unit of Primary Health Care and Medical Research Center, Oulu University Hospital, Oulu, Finland; 38 Division of Musculoskeletal and Dermatological Sciences, University of Manchester, Manchester, UK; 39 Department of Biostatistics, University of Liverpool, Liverpool, UK; 40 Wellcome Centre for Human genetics, University of Oxford, Oxford, UK; 41 Department of Drug Treatment, Sahlgrenska University Hospital, Gothenburg, Sweden; 42 Usher Institute of Population Health Sciences and Informatics, University of Edinburgh, Edinburgh, UK; 43 Department of Social and Behavioural Science, Harvard TH Chan School of Public Health, Boston, MA USA; 44 Institute for Community Medicine, University Medicine Greifswald, Walther-Rathenau-Str. 48, 17489 Greifswald, Germany; 45 Computational Biology Unit, Department of Informatics, University of Bergen, PO Box 7803, 5020 Bergen, Norway; 46 Clinical and Translational Research Institute, Newcastle University, Newcastle upon Tyne, UK; 47 Centre for Bone Research at the Sahlgrenska Academy, Department of Internal Medicine and Geriatrics, University of Gothenburg, Gothenburg, Sweden

**Keywords:** Genetic variation, Endocrinology

## Abstract

The stress hormone cortisol modulates fuel metabolism, cardiovascular homoeostasis, mood, inflammation and cognition. The CORtisol NETwork (CORNET) consortium previously identified a single locus associated with morning plasma cortisol. Identifying additional genetic variants that explain more of the variance in cortisol could provide new insights into cortisol biology and provide statistical power to test the causative role of cortisol in common diseases. The CORNET consortium extended its genome-wide association meta-analysis for morning plasma cortisol from 12,597 to 25,314 subjects and from ~2.2 M to ~7 M SNPs, in 17 population-based cohorts of European ancestries. We confirmed the genetic association with *SERPINA6/SERPINA1*. This locus contains genes encoding corticosteroid binding globulin (CBG) and α1-antitrypsin. Expression quantitative trait loci (eQTL) analyses undertaken in the STARNET cohort of 600 individuals showed that specific genetic variants within the *SERPINA6/SERPINA1* locus influence expression of *SERPINA6* rather than *SERPINA1* in the liver. Moreover, trans-eQTL analysis demonstrated effects on adipose tissue gene expression, suggesting that variations in CBG levels have an effect on delivery of cortisol to peripheral tissues. Two-sample Mendelian randomisation analyses provided evidence that each genetically-determined standard deviation (SD) increase in morning plasma cortisol was associated with increased odds of chronic ischaemic heart disease (0.32, 95% CI 0.06–0.59) and myocardial infarction (0.21, 95% CI 0.00–0.43) in UK Biobank and similarly in CARDIoGRAMplusC4D. These findings reveal a causative pathway for CBG in determining cortisol action in peripheral tissues and thereby contributing to the aetiology of cardiovascular disease.

## Introduction

Cortisol plays a vital role in adaptation to environmental stress, modulating fuel metabolism, cardiovascular homoeostasis, mood, memory and inflammation [1]. Cortisol levels vary throughout the day under the control of the hypothalamic–pituitary–adrenal (HPA) axis. Patients with tumours causing excess cortisol develop Cushing’s syndrome, characterised by a host of features including obesity, hypertension, diabetes mellitus, depression, cognitive impairment and osteoporosis with an excess mortality due primarily to cardiovascular disease [2]. Similarly, higher plasma cortisol in the population associates with hypertension, hyperglycaemia, cardiovascular disease, type 2 diabetes, cognitive dysfunction and depression, while lower cortisol associates with immunological abnormalities and post-traumatic stress disorder [3–10].

We established the CORtisol NETwork (CORNET) consortium with the initial aim of identifying genetic determinants of inter-individual variation in HPA axis function. A genome-wide association meta-analysis (GWAMA), investigating ~2.2 M SNPs in 12,597 individuals from 11 European cohorts, identified a single locus on chromosome 14 associated with morning plasma cortisol at genome-wide significance [11]. The locus spans *SERPINA6* and *SERPINA1* and influences function of corticosteroid-binding globulin (CBG, the product of *SERPINA6*), a protein that binds cortisol in the blood. However, it was unclear if the effect is mediated directly through *SERPINA6* or indirectly through the product of *SERPINA1*, α1-antitrypsin, which is involved in regulating cleavage and inactivation of CBG [12]. Using these genetic variants as a proxy for morning plasma cortisol levels in Mendelian randomisation analyses we provided evidence to suggest that cortisol is a causal risk factor for coronary heart disease, but the odds ratio was not statistically significant (OR: 1.06, 95% CI: 0.98–1.15) [13]. An independent study has confirmed recently that common variants in *SERPINA6* are associated with plasma cortisol and with coronary artery disease [14].

The genetic variants in the *SERPINA6/A1* locus explain only ~0.5% of the variance in morning plasma cortisol. Moreover, attempts to identify genetic variants associated with an alternative phenotype of salivary cortisol have not been successful [15]. Identifying additional genetic variants that explain more of the variance in cortisol could provide new insights into cortisol biology and statistical power to test the causative role of cortisol in the aetiology of other common diseases. We aimed: to identify additional specific loci influencing cortisol; to refine where in the *SERPINA6/A1* locus there is an influence on cortisol; to establish whether *SERPINA6/A1* variation influences tissue-specific expression of CBG and α1-antitrypsin; and to confirm whether high cortisol is causal in ischaemic heart disease and test if it is causal in other common diseases. To achieve these aims, we undertook an extended GWAMA analysis, with more subjects and more SNPs than the original CORNET GWAMA [11], and used the results to provide instruments for expression quantitative trait loci (eQTL) and Mendelian randomisation analyses.

## Methods

### Genome-wide association meta-analysis study

We performed a meta-analysis of genome-wide association studies of morning plasma cortisol in 25,314 subjects from 17 European population-based cohorts: CROATIA-Vis (*n*  = 886), CROATIA-Korcula (*n*  = 897), CROATIA-Split (*n * = 493), ORCADES (*n*  = 1974), Rotterdam Study (*n*  = 2870), NFBC1966 (*n*  = 1324), Helsinki Birth Cohort Study 1934–44 (*n*  = 399), ALSPAC (*n*  = 1487), PREVEND (*n*  = 1151), PIVUS (*n*  = 919), Raine Study (*n* = 860), ET2DS (*n*  = 847), MrOS-Sweden (*n*  = 969), KORA (*n* = 1651), TwinsUK (*n* = 5654), SHIP (*n * = 910) and VIKING (*n* = 2073). Characteristics of the study populations are presented in Table S1 and details of each cohort are provided in Supplementary Material. All individuals were of European ancestries. Exclusion criteria were current glucocorticoid use, pregnant or breast-feeding women, and twins (exclusion of one of each twin pair). Cortisol was measured by immunoassay in blood samples collected from study participants between 0700 and 1100 h in all cohorts except for in TwinsUK which measured cortisol using liquid chromatography-mass spectrometry. All participants provided written informed consent and studies were approved by local Research Ethics Committees and/or Institutional Review Boards.

Each study performed linear regression on *z*-scores of log-transformed morning plasma cortisol (additive genetic effects), adjusted for sex, age and cohort-specific genetic ancestry. Additional models also adjusted for smoking and body mass index. Imputation of the gene-chip results used the 1000 Genomes European population reference panel. Details of the genotyping, imputation and cohort-specific adjustment for genetic ancestry are provided in Table S2 and Supplementary Material.

Quality control was carried out on the imputed genome-wide data for all 17 studies prior to meta-analysis; this excluded all SNPs with a minor allele frequency (MAF) <0.5%, call rate <95%, and poor imputation quality (MACH R2_HAT <0.30, IMPUTE PROPER_INFO <0.60, BEAGLE INFO <0.30, as appropriate). Furthermore, only SNPs with estimates from at least four studies were included, resulting in a final number of 8,452,427 SNPs. Quantile–quantile (QQ) plots and genomic control (lambda) were used to assess evidence for population structure that was not accounted for in association analyses. Sex chromosomes were not analysed. Quality control at the study-level and meta-level was performed using EasyQC software [16].

The results from all cohorts were combined into a fixed-effects meta-analysis using Stouffer’s method with weighting of *Z*-scores proportional to the square-root of the number of individuals in each sample, using METAL software [17]. Manhattan and QQ plots visualised the results using EasyStrata software [18].

### SNP-based heritability

Linkage disequilibrium (LD) score regression exploits the relationship between SNP-phenotype association strengths and LD patterns [19]. Assuming true causal effects, the SNPs which are in higher LD with nearby SNPs are expected to have more inflated test statistics, because they are more likely to tag causal variants with stronger effects. The SNP heritability was estimated using LD score regression v1.9.0 [19]. Since imputation quality can confound LD score regression results, we restricted the analysis to a list of well-imputed SNPs, as recommended by the software authors. After applying default quality control settings, the final SNP number was 1,028,327. The genome-wide summary statistics were partitioned into functional categories using the method described by Finucane et al. [20]. More details of the partitioned heritability method can be found in the Supplementary Material.

### Genetic correlations

Genetic correlations between morning plasma cortisol and selected diseases and traits from UK Biobank were estimated using bivariate LD score regression. This technique examines the correlation structure of genetic effects of SNPs across the genome. The data processing pipeline devised by Bulik-Sullivan et al. [21] was followed using LD Hub v1.9.0 software [22].

### Gene- and pathway-based association analysis

Gene- and pathway- based associations, which assign SNPs to genes and biological pathways respectively, were performed using MAGMA and FUMA software [23, 24]. SNPs were assigned to 18,062 genes using the National Centre for Biotechnology Information build 37.3. The gene boundary was defined as the start and stop site of each gene. The European panel of the 1000 Genomes data (phase 1, release 3) was used as a reference panel to account for LD between the SNPs. A Bonferroni correction was used to control for 18,062 tests (*α* = 0.05/18 062; *P* < 2.768 × 10^−6^).

### eQTL analysis in the Stockholm Tartu Atherosclerosis Reverse Networks Engineering Task (STARNET) study

eQTL analyses were undertaken in the STARNET study which is composed of Caucasian individuals of Eastern European origin (30% female), with a confirmed diagnosis of coronary artery disease. Of these individuals 27% had diabetes, 77% had hypertension and 37% had suffered a myocardial infarction before the age of 60 years. Genotyping and RNA sequencing of seven vascular and metabolic tissue samples from 600 patients undergoing coronary artery bypass surgery was performed [25]. The tissue sets available in STARNET include; whole blood, atherosclerotic-lesion free internal mammary artery, atherosclerotic aortic root, subcutaneous fat, visceral abdominal fat, skeletal muscle, and liver. Whole blood samples were taken pre-operatively and the remaining tissue biopsies were obtained during surgery.

For the analysis, we included 580 SNPs within *SERPINA6/SERPINA1* locus on chromosome 14 identified from the CORNET plasma cortisol GWAMA, defined as within 100 Kb of *SERPINA6* (±of the transcription start/end point), with no missing data, and MAF >5%. All transcripts were annotated using the Human Genome Reference Consortium Human Build 37 (GRCh37).

Transcriptome-wide eQTL associations between selected SNPs and all genes expressed in the STARNET tissues were tested using the Kruskal–Wallis test, a non-parametric ANOVA used to determine whether sample groups originate from the same distribution that has been used previously in genetic association studies [26]. Kruskal–Wallis test statistics and *p* values were computed using the kruX algorithm [27], using available pre-processed and normalised genotype and expression data matrices [25] as input. Multiple testing correction was performed for all SNP associations with every STARNET gene in each tissue separately by calculating *q* values [28] using the qvalue package in Python. eQTL associations with *SERPINA6* were visualised using the GWAS visualisation tool LocusZoom [29]. The allelic effect of individual SNPs on *SERPINA6* was visualised by constructing boxplots for each allele.

The global tissue-specific effect on gene expression for SNPs associated with plasma cortisol at a genome-wide level of significance was depicted using Q–Q plots showing the observed transcriptome-wide SNP-gene associations against the expected uniform distribution. Deviation from the uniform distribution was tested using the Kolmogorov–Smirnov test statistic and *p* value for each SNP.

Bayes factor colocalisation analysis was performed in R using the package Coloc [30]. eQTL analysis was repeated using linear regression with the R package MatrixEQTL [31] to obtain beta values required for colocalisation. linear regression and Kruskal–Wallis *p* values were consistent (Fig. S7). For visualisation of the colocalisation event, LD with the lead SNP was calculated using the package LDlinkR [32].

### Two-sample Mendelian randomisation

The instrument for morning plasma cortisol consisted of independent (*r*^2^ < 0.3) genetic variants that reached a genome-wide level of significance (*P* < 5 × 10^−8^). To detect independent top SNPs we used the clumping function as implemented in PLINK. The European samples from the 1000 Genomes Project were used to estimate LD between SNPs. Amongst those SNPs within 1000 kb and *r*^2^ < 0.3, only the SNP with the lowest *P* value was retained.

Two-sample Mendelian randomisation [33] was used to estimate the causal effect of morning plasma cortisol on hypothetically cortisol-related diseases and traits (chronic ischaemic heart disease, myocardial infarction, diabetes mellitus, body mass index and osteoporosis) available in UK Biobank and publicly available GWAS consortia in the MR Base platform [34].

Genetic instruments for various traits/conditions were also constructed to estimate the causal effect these had on morning plasma cortisol (bidirectional Mendelian randomisation). Details of the selected disease and traits, the population, consortia details and genetic instruments are provided in Table S3. The SNP-cortisol estimate was divided by the SNP-outcome estimate (Wald ratio method [35]) and then combined using inverse variance weighting. Additional analyses described in the Supplementary Material were performed to investigate the robustness of this causal estimate and any potential pleiotropic effects.

## Results

### Genome-wide association meta-analysis

**Fig. 1:**
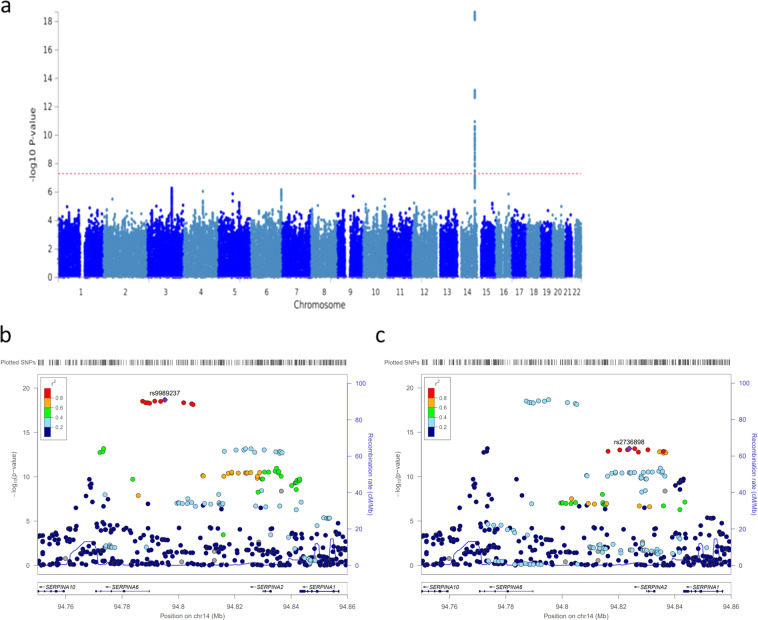
**a** Manhattan plot of −log10 *P* values of the SNP-based association analysis of morning plasma cortisol (*n* = 25,314). The locus on chr14 spans *SERPINA6* and *SERPINA1* genes; no other loci reached genome-wide significance. **b**, **c** Zoomed in Manhattan plot (LocusZoom plot) of −log10 *P* values of the SNP-based association analysis of morning plasma cortisol (*n* = 25,314). These show two (of the four) LD blocks (*r*^2^ > 0.3) in this locus

The GWAMA of morning plasma cortisol levels in 25,314 identified a single locus on chromosome 14 reaching a genome-wide level of significance (*p* < 5 × 10^−8^) (Fig. 1). This is the same locus as the CORNET consortium previously identified [11] and includes the *SERPINA6* gene, encoding corticosteroid binding globulin (CBG), and the *SERPINA1* gene, encoding α1-antitrypsin, an inhibitor of neutrophil elastase which cleaves and inactivates CBG. In an additive genetic model, the top SNP rs9989237 reported a per minor allele effect of 0.11 cortisol *z*-score (*p*  = 2.2 × 10^−19^). The effect allele frequency was 0.22 and this variation explained 0.13% of the morning plasma cortisol variance. Within this locus we identified four blocks of SNPs in low LD (*r*^2^ < 0.3) visualised in Figs. 1, S1.

### SNP-based heritability

Using LD score regression, common SNPs across the genome were found to explain 4.2% (s.e. 1.9%) of the phenotypic variation of morning plasma cortisol. There was no evidence of enrichment in a particular cell type, and particularly not in the adrenal/pancreas or liver cell type groups of SNPs, when partitioning the heritability into functional cell types (all *P* values > 0.5, Table S4).

### Genetic correlations

LD score regression was used to test whether genetic variants associated with morning plasma cortisol also contribute to health-related traits. Estimated genetic correlations are presented in Fig. S2. There was evidence of a positive genetic correlation between morning plasma cortisol and acute myocardial infarction (*r*_g_ = 0.50, 95% CI 0.04–0.97) and a negative genetic correlation with BMI (*r*_g_ = −0.32, 95% CI −0.54 to −0.10).

### Gene-based and pathway-based association analyses

Gene-based association analysis identified three genes, *SERPINA6*, *SERPINA1* and *SERPINA10*, all located on chromosome 14, that attained genome-wide significance following correction for multiple comparisons (Fig. S3). Pathway-based association analysis identified the top pathway as metabolism of lipids and lipoproteins (*P* = 1.6 × 10^−5^) (Table S5).

### Tissue-specific eQTL analyses

**Fig. 2:**
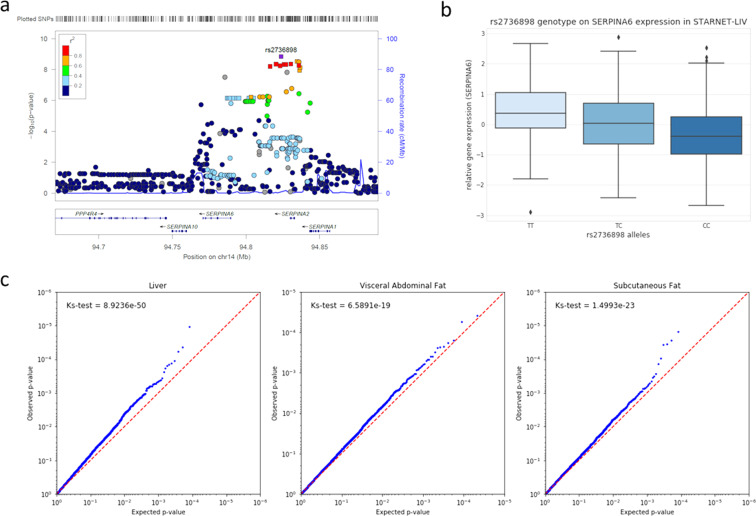
Tissue-specific association of cortisol-related SNPs with gene expression in STARNET. **a** LocusZoom plot showing genomic loci of given SNPs against measure of significance (−log10 (*p* value)) for an eQTL analysis in liver for all SNPs within 100 Kb of *SERPINA6*. Squares represent the 21 significant cis-eQTLs (*q* ≤ 0.05) that are also at genome-wide significance in CORNET (*p* ≤ 5 × 10^−8^). **b** Genotypic effect of representative SNP for LD block 2 (rs2736898) on SERPINA6 gene expression in liver. **c** Global tissue-specific effects on gene expression for rs2736898 represented as Q–Q plots for genes in liver, subcutaneous fat and visceral abdominal fat describing observed *p* values vs. those expected by chance. Deviation from expected uniform distribution described by Kolmogorov–Smirnov test *p* value (Ks-test)

The statistical effects of 580 SNPs in the *SERPINA6* region on gene expression were obtained using individual-level genotype and RNA-sequencing data from seven vascular and metabolic tissues from the STARNET study [25]. Following multiple testing correction (*q* ≤ 0.05), 32 cis-eQTLs for *SERPINA6* were identified in the liver, the only tissue where *SERPINA6* is highly expressed (Fig. 2a). Of these 32 cis-eQTLs, 21 were also at genome-wide significance in CORNET (*p* ≤ 5 × 10^−8^), and therefore also associated with variation for plasma cortisol (Table S6).

The global effect on tissue-specific gene expression of representative SNPs from each LD block was assessed using the distribution of transcriptome-wide eQTL *p* values (Figs. S4,  S5). LD block 2, represented by the SNP rs2736898, with the alternate allele C, exerted a negative effect on *SERPINA6* expression in liver (*q* = 0.00015) (Fig. 2b) and showed the strongest tissue-specific effects, particularly in visceral abdominal fat, subcutaneous fat and liver (Fig. 2c). In all cases the allele associated with higher plasma cortisol in the GWAMA was the allele associated with higher *SERPINA6* expression in STARNET (Fig. S6).

**Fig. 3:**
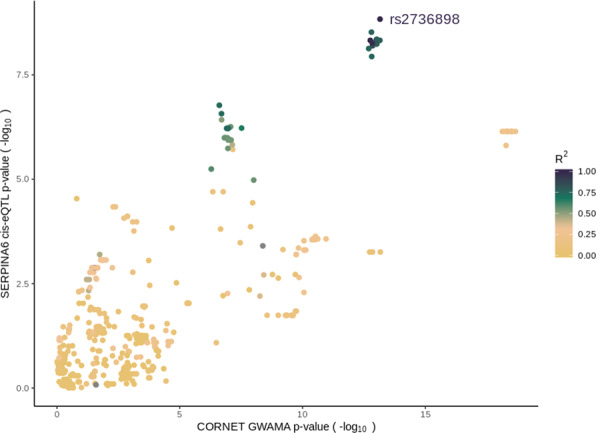
Scatterplot showing colocalisation of joint signal from CORNET GWAMA and *SERPINA6* cis-eQTLs from STARNET-liver. Includes all SNPs within 100 Kb of *SERPINA6* that were present in both datasets (*n* = 535). Colour bar indicates degree of LD with rs2736898. Formal colocalisation analysis with Coloc indicates 99.2% probability of the presence of a shared causal variant within LD block 2 mediating GWAMA and *SERPINA6* *cis*-eQTL signal

To determine if the signal identified for *SERPINA6* cis-eQTLs in liver and SNPs associated with plasma cortisol are driven by the same causal variant, Bayes factor colocalisation analysis was performed while accounting for allelic heterogeneity [30] (Fig. 3). The probability of both traits sharing a causal variant was low (40.6%) when examining all SNPs within 100 Kb of *SERPINA6*. However, when examining each LD block individually, the block represented by rs2736898 returns a 99.2% probability of shared causal variant in this region (Table S7).

### Two-sample Mendelian randomisation

**Fig. 4:**
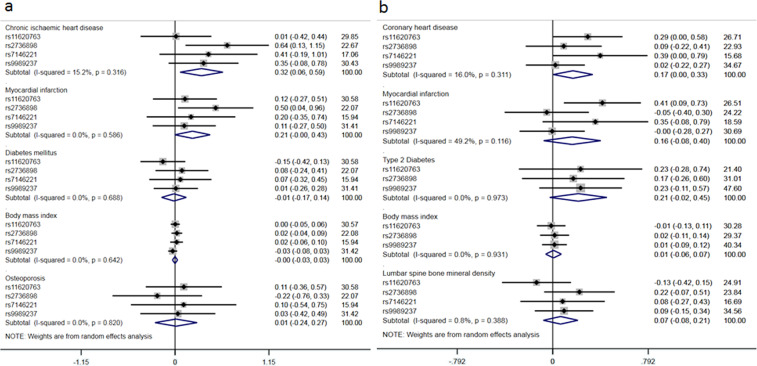
Causal estimates of a 1 SD increase in morning plasma cortisol on relevant disease and trait outcomes. Estimates are from two-sample Mendelian randomisation analyses using inverse variance weighting to combine estimates from each genetic variant. **a** Outcomes from UK Biobank (chronic ischaemic heart disease, cases = 8755, controls = 328,444; myocardial infarction, cases = 7790, controls = 328,893; diabetes mellitus, cases = 16,183, controls = 320,290; body mass index, sample size = 336,107; osteoporosis, cases = 5266, controls = 331,893); (**b**) Equivalent outcomes from non-UK Biobank sources—CARDIoGRAMplusC4D (coronary heart disease, cases = 60,801, controls = 123,504; myocardial infarction, cases = 43,676, controls = 128,199), DIAGRAM (type 2 diabetes, cases = 26,488, controls = 83,964), GIANT (BMI, sample size = 339,224) and GEFOS (lumber spine mineral density, sample size = 28,498)

The clumping procedure identified four SNPs (rs9989237, rs2736898, rs11620763, rs7146221) as markers representing genome-wide significant signals in this region. Two-sample Mendelian randomisation analyses provided evidence that each genetically-determined standard deviation (SD) increase in morning plasma cortisol was associated with an increased risk of chronic ischaemic heart disease (0.32, 95% CI 0.06–0.59) and myocardial infarction (0.21, 95% CI 0.00–0.43) in UK Biobank (Fig. 4a). Similar estimates were observed for these disease outcomes in non-UK Biobank cohorts (CARDIoGRAM plus C4D, Fig. 4b).

**Fig. 5:**
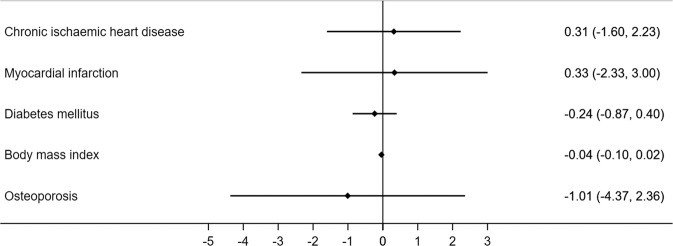
Bidirectional causal estimates of the effect of the disease or trait on morning plasma cortisol. Estimates are from two-sample Mendelian randomisation analyses using inverse variance weighting to combine estimates from each genetic variant

The bidirectional Mendelian Randomisation analyses, estimating the genetically predicted effect of various traits or disease on plasma cortisol, did not support any causal associations (Fig. 5).

## Discussion

These results confirm that genetic variation in the *SERPINA6/A1* locus on chromosome 14 is associated with morning plasma cortisol. Despite doubling the sample size and trebling the SNP density from the previous genome-wide association study [11] no new genetic loci were identified. The improved imputation of genetic markers used in this analysis allowed identification of new SNPs within the *SERPINA6/A1* locus and strengthened the instrument used in two-sample Mendelian randomisation analyses. This additional information coupled with tissue-specific gene expression data suggests that genetic variation within this locus influences expression of *SERPINA6* rather than *SERPINA1* in the liver. Furthermore, it appears that the eQTL and GWAMA signals colocalise within the block of SNPs in LD, represented by rs2736898, suggesting this block is primarily responsible for driving CBG-mediated variation for cortisol. Moreover, it demonstrates effects on adipose tissue gene expression, suggesting that resulting variations in CBG levels in turn influence the delivery of cortisol to peripheral tissues.

Given previous evidence of heritability of plasma cortisol it is surprising that the increase in sample size and SNP coverage from our earlier GWAMA did not identify any new loci associated with morning plasma cortisol. Twin studies have estimated the heritability of plasma cortisol ranging from 14 to 45% [36–38]. However, SNP heritability estimates of plasma cortisol are considerably lower at 6% [15], and were confirmed in this sample at 4%. Nevertheless, despite poor prediction of plasma cortisol by common autosomal SNPs, our interrogation of the *SERPINA6/SERPINA1* locus demonstrates important insights.

To test the functional significance of *SERPINA6/A1* variants we used eQTL analyses in the STARNET cohort. A hypothesis-free approach that investigated the effect of all 580 SNPs within the *SERPINA6/SERPINA1* locus on expression of all STARNET genes in each of the seven tissue types identified cis-eQTLs for *SERPINA6* expression in the liver, the only tissue where *SERPINA6* is highly expressed. This effect was refined to one of the LD blocks, represented by rs2736898. Importantly, genetic variation in *SERPINA6/SERPINA1* was also associated in trans-eQTL analyses with gene expression in visceral abdominal fat and subcutaneous fat as well as liver. These results suggest that not only does cortisol-associated genetic variation influence CBG expression in the liver, but it also influences cortisol signalling in peripheral tissues, an effect that is likely to be mediated by CBG.

CBG binds ~90% of cortisol in plasma but it is usually thought that only the free cortisol can access tissues and have biological effects. Patients with mutations in *SERPINA6* [39] and animals with deletion of CBG, however, exhibit features consistent with cortisol deficiency despite biochemical changes in the CBG-bound rather than free cortisol pool. Our data support the hitherto speculative evidence that CBG is involved actively in delivery of cortisol to peripheral tissues. We and others had proposed that this is mediated by altered cleavage of CBG by neutrophil elastase in tissues [11, 12], and evidence from immunoassays suggested that variants in *SERPINA6/SERPINA1* might mediate their effect through altered inhibition of neutrophil elastase by α1-antitrypsin. However, the more detailed analyses facilitated by the expanded GWAMA presented here do not support this interpretation since we did not identify eQTLs for α1-antitrypsin expression. Moreover, the reliability of immunoassays to determine CBG cleavage has since been called into question [40].

Epidemiological analysis of cortisol has demonstrated associations with a large number of diseases and traits [2–9], but the direction of causality, if any, has not been established. The improved imputation of genetic markers used in this analysis allowed identification of new SNPs within the *SERPINA6/A1* locus and strengthened the instrument used in the two-sample Mendelian randomisation analyses. We tested whether genetically-elevated cortisol is causally associated with cortisol-related outcomes selected from the features of Cushing’s syndrome—a rare condition caused by tumours secreting ACTH or cortisol—that are highly prevalent in the general population. Consistent with previous reports based on a less refined genetic instrument [13, 14] we found evidence that cortisol causally increases the risk of heart disease. However, we did not find conclusive evidence that elevated cortisol causes type 2 diabetes, osteoporosis or obesity; given the relatively small variance in cortisol accounted for by the genetic instrument, it is possible that causal associations with additional diseases and traits would be revealed by analysis of larger sample sizes. We also tested whether common diseases and traits underlie elevated plasma cortisol, using bidirectional Mendelian randomisation analyses. The strongest evidence suggested that higher BMI reduces cortisol levels, consistent with prior epidemiological and experimental evidence that obesity enhances clearance of cortisol from the circulation [1].

It is arguable that morning plasma cortisol or salivary cortisol are poor surrogates for overall cortisol exposure given their diurnal and ultradian fluctuations and variation with acute stress. A more robust assessment of cortisol phenotype, perhaps from hair cortisol [41] or ambulatory sampling of interstitial fluid over 24 h, may reveal stronger epidemiological and genetic associations.

In summary, this large GWAMA has revealed the limited magnitude and range of genetic effects on plasma cortisol, but has identified a pathway from variation in the *SERPINA6/SERPINA1* locus through variation in liver *SERPINA6* expression to variation in CBG-mediated gene transcription including in adipose tissue that is causally associated with cardiovascular disease.

## Supplementary information

Table S1

Table S2

Table S3

Table S4

Table S5

Table S6

Table S7

Figure S1

Figure S2

Figure S3

Figure S4

Figure S5

Figure S6

Figure S7

full supp list
