# Supplementary material for: Variation in the SERPINA6/SERPINA1 locus alters morning plasma cortisol, hepatic corticosteroid binding globulin expression, gene expression in peripheral tissues, and risk of cardiovascular disease
Source: J Hum Genet. 2021 Jan 20;66(6):625–36. doi: 10.1038/s10038-020-00895-6 (PMC8144017; doi:10.1038/s10038-020-00895-6)
Supplement: Supplementary file 1 — Table S1 [file 10038_2020_895_MOESM1_ESM.pdf]

Cohort	N	Male (%)	Age in years		Plasma cortisol in nmol/l		Sampling time
			Mean (sd)	Range	Mean (sd)	Range	
ORCADES	1974	45.4	53.5 (15.7)	17-97	765 (315)	11-3641	0830-1030
CROATIA-Korcula	898	36.2	56.2 (13.9)	18-98	698 (207)	59-815	0800-0900
CROATIA-Split	496	42.9	45.0 (14.7)	18-85	979 (404)	94-2831	0730-0900
CROATIA-Vis	892	43.5	56.4 (15.5)	18-93	622 (230)	64-1820	0730-0900
Rotterdam Study	6497	43.6	63.3 (9.6)	45.5-106	359 (115)	14-966	0800-1100
HBCS1934-44	451	36.1	60.61 (2.80)	56 - 67	393 (120)	125-990	0750-1055
NFBC1966	1324	0	31.1 (0.3)	30.3-32.4	380 (160)	40-2370	0800-1100
ALSPAC	1,567	50.3	15.43 (0.26)	14-17	486 (174)	58-1683	0800-1057
PIVUS	919	50.2	70.2 (0.17)	69-72	386 (125)	31-930	0800-1000
PREVEND	1151	50.6	49.4 (13.0)	28-75	442 (201)	20-1734	0800-1100
ET2DS	1048	51.3	67.9 (4.2)	60-74	731 (190)	102-1447	0800-0830
Raine Study	860	51.9	17.1 (0.29)	16-18	614 (235)	36-1654	Awakening (before 1000)
MrOS Sweden	969	100	75.3 (3.2)	70-81	487 (133)	70-1550	0700-1000
VIKING	2073	39.9	49.9 (15.2)	18-91	292 (170)	7-1885	0800-1030
SHIP	910	44.7	49.8 (13.8)	20-81	*	*	Before 1300
TwinsUK	5654	0	53.3 (13.8)	18-84	*	*	0800-1200
KORA	1651	48.6	60.92 (8.7)	31-77	*	*	N/A
