# Supplementary material for: Variation in the SERPINA6/SERPINA1 locus alters morning plasma cortisol, hepatic corticosteroid binding globulin expression, gene expression in peripheral tissues, and risk of cardiovascular disease
Source: J Hum Genet. 2021 Jan 20;66(6):625–36. doi: 10.1038/s10038-020-00895-6 (PMC8144017; doi:10.1038/s10038-020-00895-6)
Supplement: Supplementary file 2 — Table S2 [file 10038_2020_895_MOESM2_ESM.pdf]

Cohort	Genotyping platform	Calling Algorithm	Reference Panel
ORCADES	Illumina HumanHap300	MACH	Haplotype Reference Consortium
CROATIA-Korcula	Illumina HumanCNV370	MACH	1000 Genomes Phase 3
CROATIA-Split	Illumina HumanCNV370	MACH	1000 Genomes Phase 3
CROATIA-Vis	Illumina HumanHap300	MACH	1000 Genomes Phase 3
Rotterdam Study	Illumina HumanHap 550v3 and Illumina HumanHap 610	MACH	1000 Genomes Phase 3
HBCS1934-44	Modified Illumina 610k	MACH	1000 Genomes Phase 3
NFBC1966	Illumina HumanCNV370DUO	IMPUTE	1000 Genomes Phase 3
ALSPAC	Illumina HumanHap550K	MACH	1000 Genomes Phase 3
PIVUS	merged Human Omni Express and MetaboChip	IMPUTE2	1000 Genomes Phase 3
PREVEND	Illumina Cyto SNP12 v2 array	Beagle 3.3.1	1000 Genomes Phase 3
ET2DS	Illumina HumanHap300	MACH	1000 Genomes Phase 3
Raine Study	Illumina Human660W-Quad	MACH	1000 Genomes Phase 3
MrOS Sweden	Illumina HumanOmni1_Quad_v1-0 B array	IMPUTE2	1000 Genomes Phase 3
VIKING	Illumina HumanOmniExpressExome8v1-2_A	MACH	1000 Genomes Phase 3
SHIP	Affymetrix 6.0	Impute	1000 Genomes Phase 3
TwinsUK	Illumina HumanHap300	MACH	1000 Genomes Phase 3
KORA	Affymetrix 6.0	Impute	1000 Genomes Phase 3
