# Supplementary material for: Variation in the SERPINA6/SERPINA1 locus alters morning plasma cortisol, hepatic corticosteroid binding globulin expression, gene expression in peripheral tissues, and risk of cardiovascular disease
Source: J Hum Genet. 2021 Jan 20;66(6):625–36. doi: 10.1038/s10038-020-00895-6 (PMC8144017; doi:10.1038/s10038-020-00895-6)
Supplement: Supplementary file 3 — Table S3 [file 10038_2020_895_MOESM3_ESM.pdf]

Exposure	Cases	Controls	Sample size	Number of SNPs used in instrument
Osteoporosis	5266	331893	337159	8
Body mass index			336107	287
Diabetes mellitus	16183	320290	336473	44
Myocardial infarction	7790	328893	336683	11
Chronic ischaemic heart disease	8755	328444	337199	15
