# Supplementary material for: Variation in the SERPINA6/SERPINA1 locus alters morning plasma cortisol, hepatic corticosteroid binding globulin expression, gene expression in peripheral tissues, and risk of cardiovascular disease
Source: J Hum Genet. 2021 Jan 20;66(6):625–36. doi: 10.1038/s10038-020-00895-6 (PMC8144017; doi:10.1038/s10038-020-00895-6)
Supplement: Supplementary file 4 — Table S4 [file 10038_2020_895_MOESM4_ESM.pdf]

Cell Type	Prop._SNPs	Prop._h2	Prop._h2_std_error	Enrichment	Enrichment_std_error	Enrichment_p
Adrenal_Pancreas	0.09	1.63	2.82	17.43	30.14	0.59
Cardiovascular	0.11	0.55	1.78	4.95	16.05	0.81
CNS	0.15	0.84	2.02	5.63	13.56	0.73
Connective_Bone	0.11	0.86	1.94	7.52	16.87	0.70
GI	0.17	2.35	3.74	14.01	22.32	0.56
Immune	0.23	-0.14	1.84	-0.61	7.87	0.84
Kidney	0.04	1.92	2.88	45.03	67.65	0.52
Liver	0.07	2.77	4.63	38.38	64.17	0.56
Other	0.20	-0.01	2.10	-0.05	10.35	0.92
SkeletalMuscle	0.10	-0.05	1.62	-0.45	15.60	0.93
