# Supplementary material for: Variation in the SERPINA6/SERPINA1 locus alters morning plasma cortisol, hepatic corticosteroid binding globulin expression, gene expression in peripheral tissues, and risk of cardiovascular disease
Source: J Hum Genet. 2021 Jan 20;66(6):625–36. doi: 10.1038/s10038-020-00895-6 (PMC8144017; doi:10.1038/s10038-020-00895-6)
Supplement: Supplementary file 5 — Table S5 [file 10038_2020_895_MOESM5_ESM.pdf]

Pathway name	P value
REACTOME_METABOLISM_OF_LIPIDS_AND_LIPOPROTEINS	1.35E-05
REACTOME_SPHINGOLIPID_METABOLISM	1.65E-04
KEGG_ALPHA_LINOLENIC_ACID_METABOLISM	2.61E-04
BIOCARTA_EGFR_SMRTE_PATHWAY	3.65E-04
REACTOME_PHOSPHOLIPID_METABOLISM	5.13E-04
REACTOME_VOLTAGE_GATED_POTASSIUM_CHANNELS	6.73E-04
REACTOME_GLYCOSPHINGOLIPID_METABOLISM	1.42E-03
KEGG_TGF_BETA_SIGNALING_PATHWAY	1.73E-03
REACTOME_BETA_DEFENSINS	2.41E-03
KEGG_STARCH_AND_SUCROSE_METABOLISM	2.51E-03
REACTOME_CIRCADIAN_CLOCK	2.73E-03
REACTOME_BMAL1_CLOCK_NPAS2_ACTIVATES_CIRCADIAN_EXPRESSION	3.21E-03
REACTOME_DEFENSINS	3.64E-03
REACTOME_CIRCADIAN_REPRESSION_OF_EXPRESSION_BY_REV_ERBA	4.60E-03
KEGG_SPHINGOLIPID_METABOLISM	4.66E-03
