# Supplementary material for: Variation in the SERPINA6/SERPINA1 locus alters morning plasma cortisol, hepatic corticosteroid binding globulin expression, gene expression in peripheral tissues, and risk of cardiovascular disease
Source: J Hum Genet. 2021 Jan 20;66(6):625–36. doi: 10.1038/s10038-020-00895-6 (PMC8144017; doi:10.1038/s10038-020-00895-6)
Supplement: Supplementary file 6 — Table S6 [file 10038_2020_895_MOESM6_ESM.pdf]

SNP	Genomic Loci	Kruskal Wallis	p-value	q-value	CORNET p-value
rs2736898	14:94823817	40.68215	1.47E-09	0.000153	7.03E-14
rs3762132	14:94834575	39.24606	3.00E-09	0.000305	1.57E-13
rs59036614	14:94830448	38.45662	4.46E-09	0.000441	9.49E-14
rs2749529	14:94820459	38.41395	4.56E-09	0.000445	9.92E-14
rs2749527	14:94827068	38.34012	4.73E-09	0.000445	1.75E-13
rs2013150	14:94825769	38.34473	4.72E-09	0.000445	7.12E-14
rs941594	14:94835914	38.04932	5.47E-09	0.000508	1.45E-13
rs2736899	14:94823220	37.93856	5.78E-09	0.000522	9.51E-14
rs2749530	14:94816299	37.74902	6.35E-09	0.000551	1.40E-13
rs1243171	14:94836784	37.44135	7.41E-09	0.000635	2.02E-13
rs1243173	14:94836298	36.55909	1.15E-08	0.000947	1.53E-13
rs2749539	14:94803365	28.66661	5.96E-07	0.034184	3.04E-08
rs4491436	14:94804700	28.2968	7.17E-07	0.0381	5.97E-19
rs718187	14:94801860	28.2968	7.17E-07	0.0381	4.52E-19
rs9989237	14:94795202	28.2968	7.17E-07	0.0381	2.16E-19
rs12589136	14:94793686	28.2968	7.17E-07	0.0381	3.23E-19
rs6575415	14:94791601	28.2968	7.17E-07	0.0381	2.97E-19
rs2281518	14:94789117	28.2968	7.17E-07	0.0381	4.58E-19
rs941599	14:94788341	28.2968	7.17E-07	0.0381	4.41E-19
rs4905187	14:94805193	28.2968	7.17E-07	0.0381	7.34E-19
rs7161521	14:94787288	28.2968	7.17E-07	0.0381	3.07E-19
