# Supplementary material for: Variation in the SERPINA6/SERPINA1 locus alters morning plasma cortisol, hepatic corticosteroid binding globulin expression, gene expression in peripheral tissues, and risk of cardiovascular disease
Source: J Hum Genet. 2021 Jan 20;66(6):625–36. doi: 10.1038/s10038-020-00895-6 (PMC8144017; doi:10.1038/s10038-020-00895-6)
Supplement: Supplementary file 7 — Table S7 [file 10038_2020_895_MOESM7_ESM.pdf]

LD_block	nsnps	PP.H0.abf	PP.H1.abf	PP.H2.abf	PP.H3.abf	PP.H4.abf
LD1	4	4.16E-05	7.82E-07	0.259387	0.004138	0.736433
LD2	13	4.81E-14	1.55E-10	2.92E-06	0.008404	0.991593
LD3	2	5.14E-05	1.34E-06	0.047832	0.000298	0.951817
LD4	52	2.24E-17	5.87E-16	0.00038	0.008972	0.990648
All_SNPs	535	9.76E-18	3.48E-14	0.000167	0.594	0.406
