# Supplementary figures and images for: Variation in the SERPINA6/SERPINA1 locus alters morning plasma cortisol, hepatic corticosteroid binding globulin expression, gene expression in peripheral tissues, and risk of cardiovascular disease
Source: J Hum Genet. 2021 Jan 20;66(6):625–36. doi: 10.1038/s10038-020-00895-6 (PMC8144017; doi:10.1038/s10038-020-00895-6)

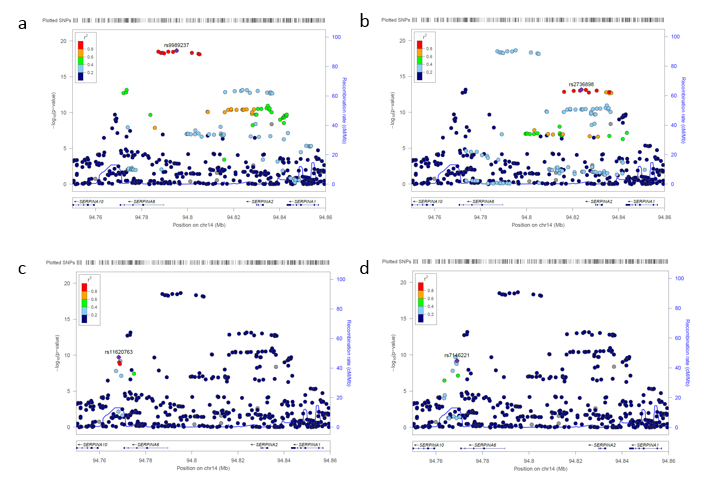

Supplement: Supplementary file 8 — Figure S1 [file 10038_2020_895_MOESM8_ESM.tif]

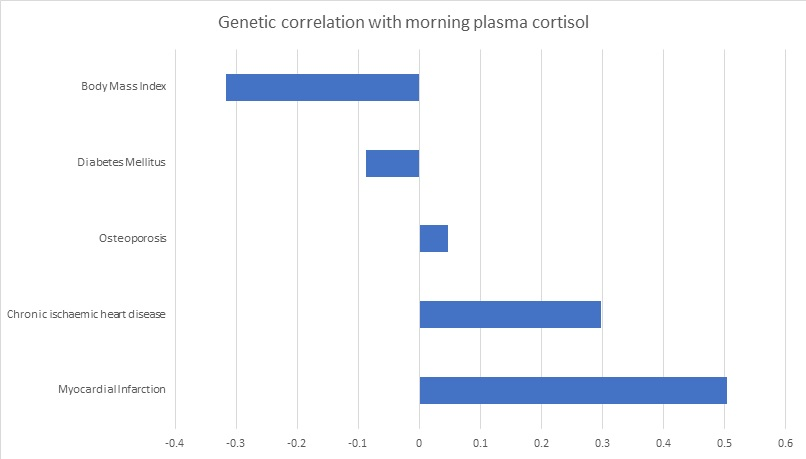

Supplement: Supplementary file 9 — Figure S2 [file 10038_2020_895_MOESM9_ESM.tif]

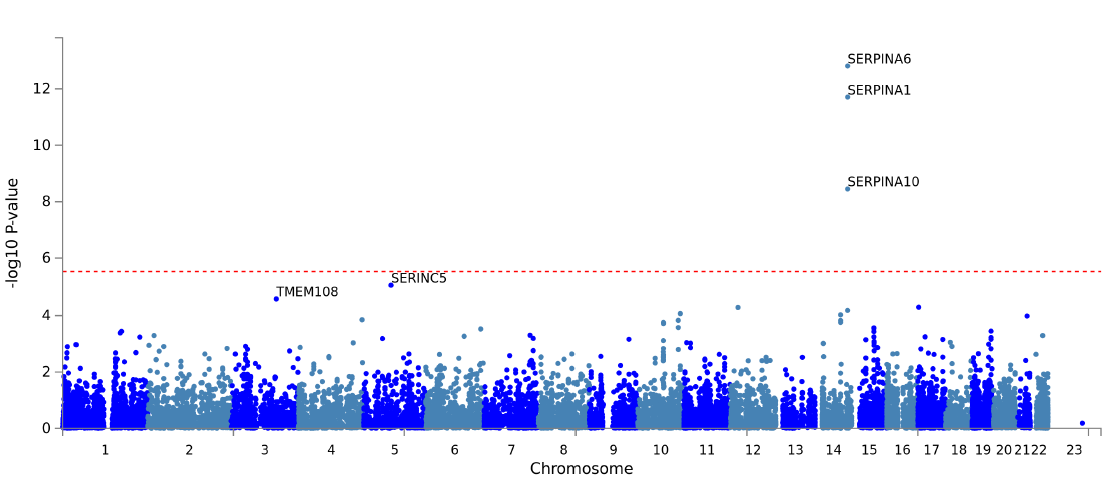

Supplement: Supplementary file 10 — Figure S3 [file 10038_2020_895_MOESM10_ESM.tif]

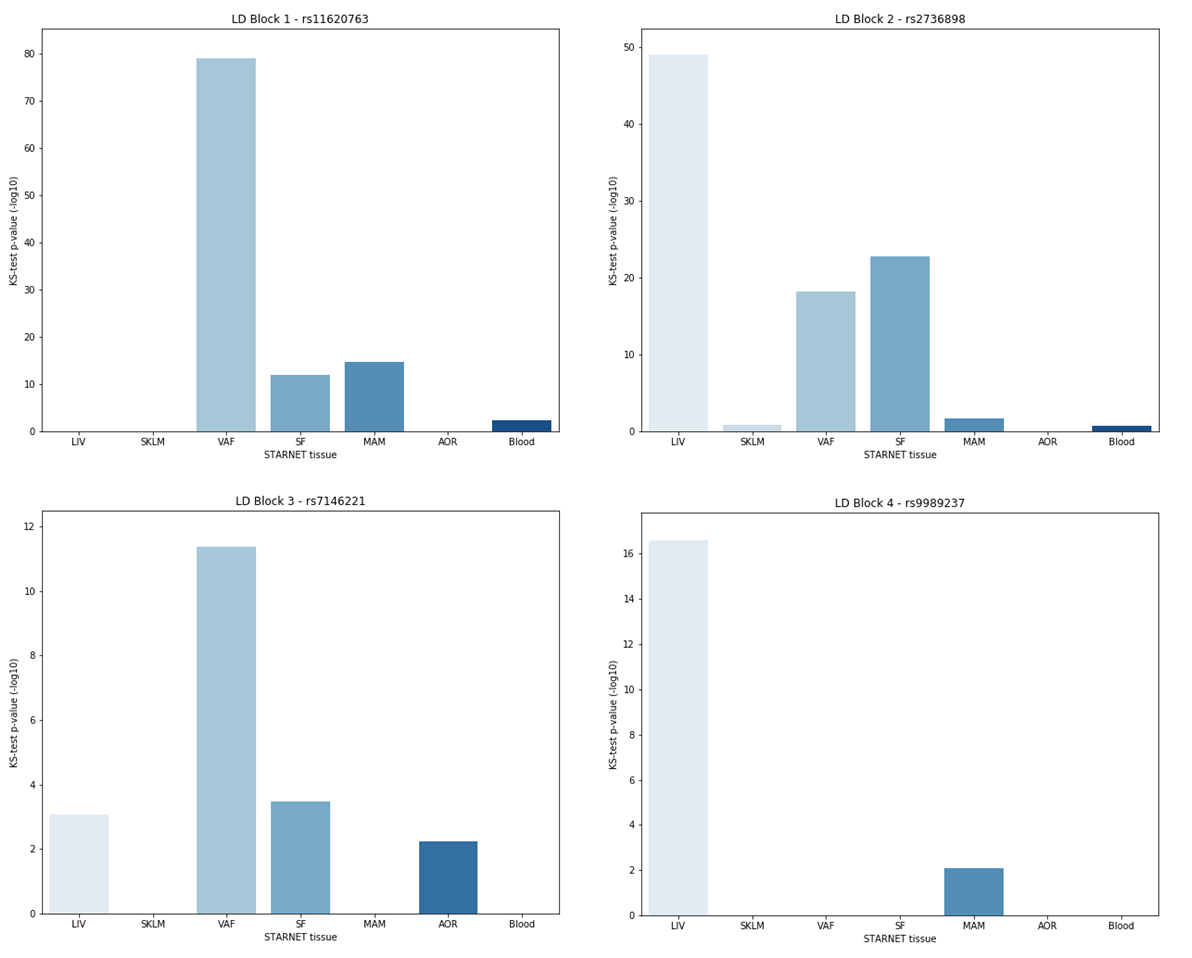

Supplement: Supplementary file 11 — Figure S4 [file 10038_2020_895_MOESM11_ESM.tif]

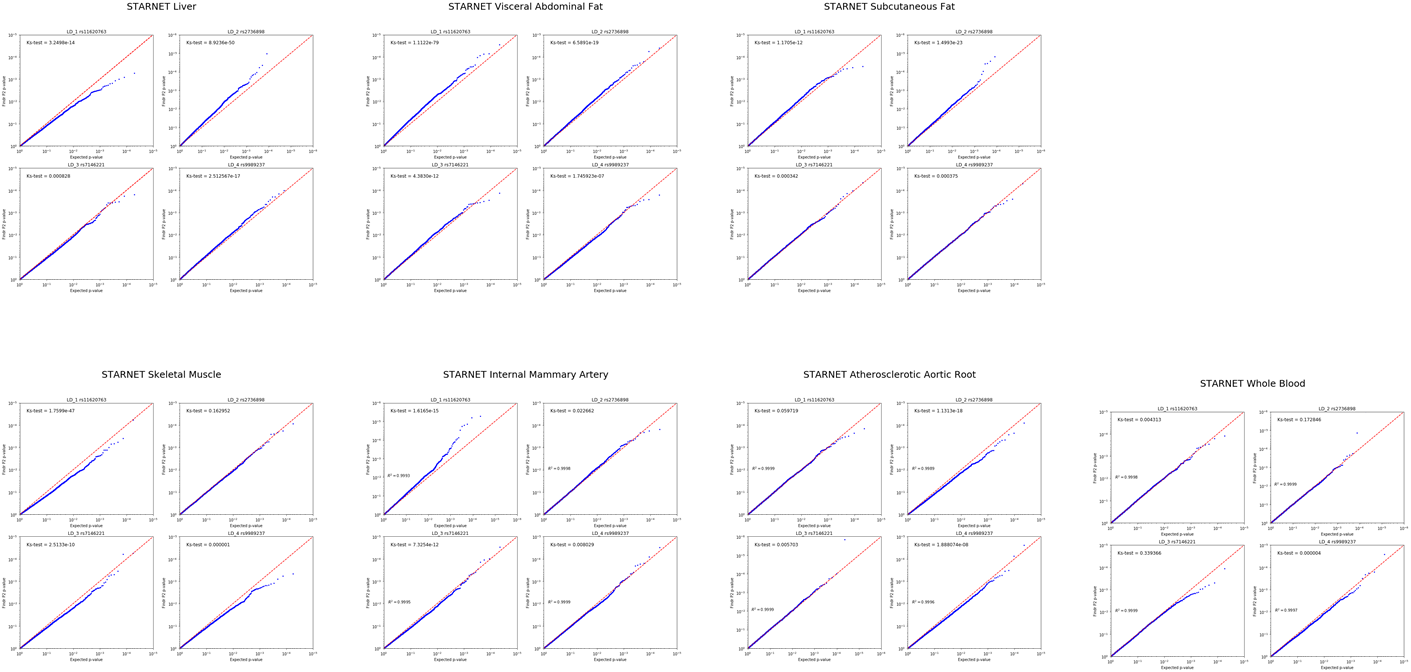

Supplement: Supplementary file 12 — Figure S5 [file 10038_2020_895_MOESM12_ESM.tif]

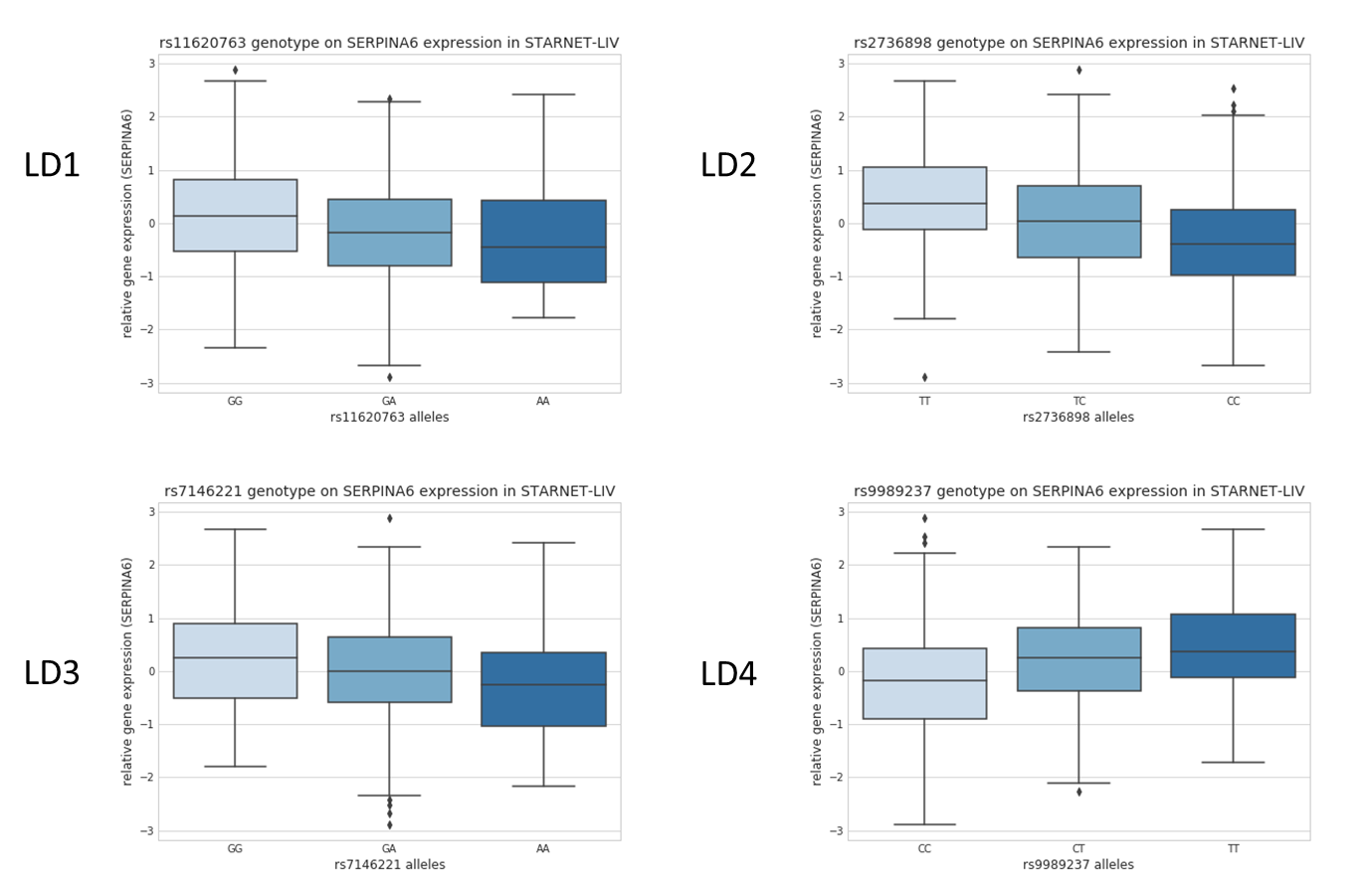

Supplement: Supplementary file 13 — Figure S6 [file 10038_2020_895_MOESM13_ESM.tif]

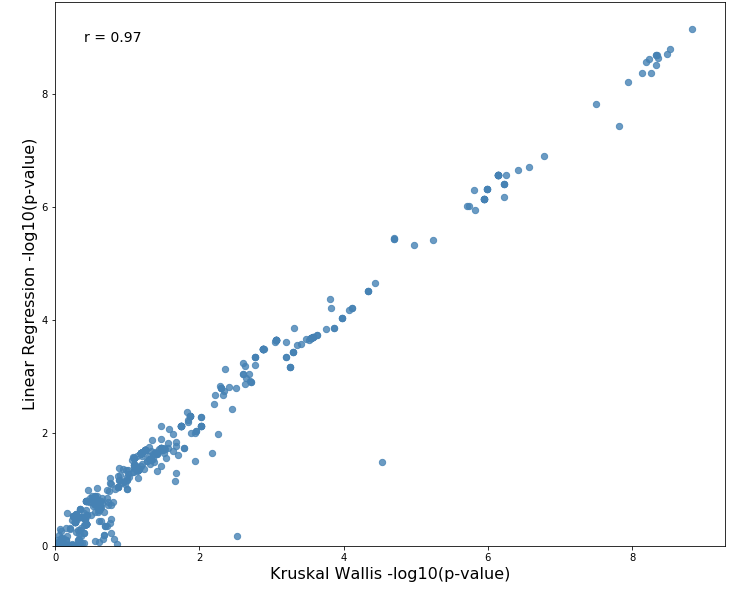

Supplement: Supplementary file 14 — Figure S7 [file 10038_2020_895_MOESM14_ESM.tif]
