# Supplementary material for: Variation in the SERPINA6/SERPINA1 locus alters morning plasma cortisol, hepatic corticosteroid binding globulin expression, gene expression in peripheral tissues, and risk of cardiovascular disease
Source: J Hum Genet. 2021 Jan 20;66(6):625–36. doi: 10.1038/s10038-020-00895-6 (PMC8144017; doi:10.1038/s10038-020-00895-6)
Supplement: Supplementary file 15 — full supp list [file 10038_2020_895_MOESM15_ESM.docx]

**Supplemental material**

**Descriptions of individual cohorts contributing to the CORNET consortium**

*ORCADES Study*

The Orkney Complex Disease Study (ORCADES) is a study based in Orkney, Scotland designed to examine genetic determinants of numerous quantitative phenotypic traits. The study population of n=2078, aged 17-100 years was recruited from a genetic isolated population resource between 2005-2011. Plasma samples were collected after overnight fast between 0830 and 1030 h. Plasma cortisol was measured by radioimmunoassay (RIA) (MP Biomedicals, Cambridge, UK).

*Croatia-Korcula study*

The CROATIA-Korcula study, Croatia, is a family-based, cross-sectional study in the isolated island of Korcula that included 965 examinees aged 18-95. Blood samples were collected in 2007 along with many clinical and biochemical measures and lifestyle and health questionnaires. Plasma cortisol was measured by RIA (MP Biomedicals, Cambridge, UK).

*Croatia-Split study*

The CROATIA-Split study, Croatia, is a population-based, cross-sectional study in the Dalmatian City of Split that so far includes 1000 examinees aged 18-95. Blood samples were collected in 2009-2011 along with many clinical and biochemical measures and lifestyle and health questionnaires. Plasma cortisol was measured by RIA (MP Biomedicals, Cambridge, UK).

*Croatia-Vis study*

The CROATIA-Vis study, Croatia, is a family-based, cross-sectional study in the isolated island of Vis that included 1,056 examinees aged 18-93. Blood samples were collected in 2003 and 2004 along with many clinical and biochemical measures and lifestyle and health questionnaires. Plasma cortisol was measured by RIA (MP Biomedicals, Cambridge, UK).

*Rotterdam study*

The Rotterdam Study (RS) is an ongoing population-based cohort study of risk factors for chronic diseases in the elderly which includes approximately 8,000 participants who live in Rotterdam. Detailed information on design, objectives and methods has been presented elsewhere[1]. The third study wave (1997-1999) was used in the current study (N=4797). In total, 2945 had serum cortisol levels and genotype data. The Medical Ethics Committee of the Erasmus Medical Centre approved the Rotterdam Study and written informed consent was obtained from all participants. Cortisol was measured by RIA (DPC, Los Angeles, CA, USA).

*Helsinki Birth Cohort Study 1934-1944*

The Helsinki Birth Cohort Study (HBCS) is composed of 8,760 individuals born between the years 1934-44 in one of the two main maternity hospitals in Helsinki, Finland. Between 2001 and 2003, a randomly selected sample of 928 males and 1075 females participated in a clinical follow-up visit with a focus on cardiovascular, metabolic and reproductive health, cognitive function and depressive symptoms. Blood samples were collected in the visit along with various other biochemical and questionnaire-based data. There were 451 women and men (36.1% men) with valid genotype and phenotype data. The mean age of the participants was 60.6 years (SD=2.8). The HBCS research protocol was approved by the Institutional Review Board of the National Public Health Institute and all participants have signed an informed consent. Cortisol was measured on an immunoanalyser (Bayer Inc., Garytown, NY, USA)

*Northern Finland Birth Cohort 1966 (NFBC1966)*

The Northern Finland Birth Cohort 1966 study was initiated in 1965 by enrolling mothers living in the two Northernmost provinces of Finland (Oulu and Lapland) and with expected dates of delivery in 1966[2]. Altogether 12,231 children were born into the cohort, 12,058 of them live-born. The original data have been supplemented by data collected with postal questionnaires at the ages of 1, 14 and 31 years and various hospital records and national register data. At 31 years of age, those living in the original target area (Northern Finland) or in the capital (Helsinki) area were invited to a clinical examination, in which 71% (N=6033) participated. Blood samples were drawn and DNA was extracted successfully for 5753 subjects. Serum cortisol was analysed for a subset of the study participants (N=1192). All participants gave written informed consent and ethical committees of University of Oulu and the Northern Ostrobothnia Hospital District have approved the study. Cortisol was measured by RIA (Orion Diagnostica, Oulunsalo, Finland).

*Avon Longitudinal Study of Parents and Children (ALSPAC)*

ALSPAC recruited pregnant women resident in Avon, UK with expected dates of delivery 1st April 1991 to 31st December 1992. In approximately 1998, the initial sample was expanded by recruiting eligible cases who did not join the study originally. Further details are published previously [3]. The total sample size for analyses using any data collected after the age of seven is from 15,247 pregnancies, resulting in 14,701 children alive at 1 year of age. Samples for the studies reported here were obtained from 1,567 for participants who had attended before 1100h. All the data are available through a fully searchable data dictionary at <http://www.bris.ac.uk/alspac/researchers/data-access/data-dictionary>. Ethical approval for the study was obtained from the ALSPAC Ethics and Law Committee and the Local Research Ethics Committees. Plasma cortisol was measured by RIA (MP Biomedicals, Cambridge, UK).

*PIVUS study*

The participants in the Prospective Investigation of the Vasculature in Uppsala Seniors (PIVUS) study were randomly sampled from all men and women at age 70 living in Uppsala County in 2001. Of the 2025 individuals invited, 1016 participated. The participants underwent a medical examination including a detailed questionnaire on lifestyle and socioeconomic factors, fasting blood sampling, blood pressure measurement and anthropometric measurements. Blood and plasma samples have been frozen until analysis, and blood tests performed include a wide variety of traditional and more recent CVD risk factors, along with DNA extraction. Cortisol was measured on an immunoanalyser (Modular E170, Roche Diagnostics, Mannheim, Germany).

*PREVEND study*

The Prevention of Renal and Vascular End-stage Disease (PREVEND) study is an ongoing prospective study investigating the natural course of increased levels of urinary albumin excretion and its relation to renal and cardiovascular disease. Details of the protocol are described at [www.prevend.org](http://www.prevend.org/). Cortisol was measured on an Abbot Axsym analyser using a Fluorescence Polarization Immunoassay (FPIA)

*Edinburgh Type 2 Diabetes Study (ET2DS)*

The ET2DS recruited a randomly-selected cohort of 1066 men and women aged 60-74 years with type 2 diabetes resident in Lothian, UK. Plasma samples and DNA used in the current analysis were collected on all participants in 2006/7. Further details have been published previously[4]. Plasma cortisol was measured by RIA (MP Biomedicals, Cambridge, UK).

*The Raine Study*

The Raine Study recruited approximately 2,900 pregnancies from King Edward Memorial Hospital between 1989 and 1991 as part of a randomised controlled trial which evaluated repeated ultrasounds in pregnancy. Detailed data were collected throughout pregnancy and at follow-ups carried out at ages 1, 2, 3, 6, 8, 10, 14, 17, 20, and 22 years. Between 2006 and 2009, specimens were collected from 1,408 study participants in the home environment as part of the 17-year follow-up. Study participants received a home visit from a research nurse on weekdays and fasted blood was drawn shortly after awakening; all samples were collected before 1000h. Cortisol was measured by RIA (GamaCoat cortisol RIA, DiaSorin, MN, USA).

*MrOS-Sweden study*

The Osteoporotic Fractures in Men (MrOS) study is a multicenter, prospective study including 3,014 elderly men in Sweden, Hong Kong (~2,000), and the United States (~6,000). The MrOS Sweden cohort consist of three sub-cohorts from three different Swedish cities (n=1,005 in Malmo, n=1,010 in Göteborg, and n=999 in Uppsala). In this study, only participants from Göteborg were used. Study subjects were randomly identified using national population registers, contacted and asked to participate. To be eligible for the study, the subjects had to be able to walk without assistance, provide self-reported data, and sign an informed consent; there were no other exclusion criteria. The study was approved by the ethics committee at the University of Gothenburg. Informed consent was obtained from all study participants. Cortisol was measured by immunoanalyser (Elecsys, Roche Diagnostics Scandinavia AB).

*KORA*

The Cooperative Health Research in the Augsburg Region (KORA) is a representative random sample of all inhabitants in the Augsburg region between 25 and 74 years. Since 1984 there have been four waves of initial medical examinations at intervals of five years. In total nearly 20,000 participants have been questioned at regular intervals with regard to their health condition and physical examination. This makes it possible to show the progress of a disease over periods of up to more than 20 years.

*TwinsUK*

TwinsUK is the UK’s largest adult twin registry and the most clinically detailed in the world. Set up in 1992, there are now almost 14000 identical and non-identical twins from across the UK, with ages between sixteen and one hundred. TwinsUK aims to investigate the genetic and environmental basis of a range of complex diseases and conditions.

*SHIP*

The Study of Health in Pomerania (SHIP) is a population-based project conducted in Northeast Germany. The main purpose is to assess prevalence and incidence of common risk factors, subclinical disorders and clinical diseases, and to investigate associations and interactions among them. The first SHIP cohort included 4,308 individuals at baseline (SHIP-0, response 68.8%) and 3,300 after 5 years (SHIP-1, response 83.6%).

*VIKING*

The Viking Health Study - Shetland (VIKING) is a family-based, cross-sectional study that seeks to identify genetic factors influencing cardiovascular and other disease risk in the population isolate of the Shetland Isles in northern Scotland. Genetic diversity in this population is decreased compared to mainland Scotland, consistent with the high levels of endogamy historically. 2105 participants were recruited between 2013 and 2015, each having at least three grandparents from Shetland. Fasting blood samples were collected and many health-related phenotypes and environmental exposures were measured in each individual. The Shetland VHSS participants’ DNA was genotyped using the Illumina HumanOmniExpressExome8v1-2_A and genotyping quality controls and imputation carried out as described for ORCADES.

**Partitioned heritability**

The morning plasma cortisol GWAMA summary statistics were portioned into functional categories using the method developed by Finucane *et al.*[5]*.* The mean chi[6] statistic was 1.007. A mean chi[6] statistic less than 1.02 indicates there is very little polygenic signal for use with stratified LD score regression. No functional cell type was enriched (Table S4).

**Two sample Mendelian randomization methods**

This approach assumes that the gene-exposure and gene-outcome associations are estimated in non-overlapping samples and are representative of the same population (similar age, sex distribution and the same ethnic group).[6] We are unable to rule out the possibility that individuals may have participated in multiple studies and so may have contributed to CORNET and to UK Biobank. If this is the case then estimates from the two-sample Mendelian randomization analyses may be biased towards the estimate obtained from conventional methods (e.g. multivariable regression).[7]

We ran three additional analyses: first, a weighted median approach[8] which is consistent even when up to half of the information comes from invalid instrumental variables; second, a maximum likelihood approach which uses linear relationship between the risk factor and outcome and a bivariate normal distribution for the genetic association estimates; finally, inverse variant weighting (IVW) to combine each of the three SNPs which is a linear regression analysis through the mean SNP-exposure and SNP-outcome results that is forced to go through zero (i.e. constrained to have intercept zero)[9]. As a sensitivity analysis to explore horizontal pleiotropy we used MR-Egger regression[9], which is similar to IVW but does not constrain the regression line to go through zero. A non-zero intercept in MR-Egger suggests possible horizontal pleiotropy; the slope can be interpreted as the effect having relaxed the horizontal pleiotropy assumption.

**Table S1. Characteristics of participants in cohorts included in the genome-wide association meta-analysis for morning plasma cortisol**

* Cortisol measured as part of metabolite panel, therefore relative intensity is measured and not absolute concentration.

|  |  |  | Age in years | |  | Plasma cortisol in nmol/l | |  |
| --- | --- | --- | --- | --- | --- | --- | --- | --- |
| Cohort | N | Male (%) | Mean (sd) | Range |  | Mean (sd) | Range | Sampling time |
| ORCADES | 1974 | 45.4 | 53.5 (15.7) | 17-97 |  | 765 (315) | 11-3641 | 0830-1030 |
| CROATIA-Korcula | 898 | 36.2 | 56.2 (13.9) | 18-98 |  | 698 (207) | 59-815 | 0800-0900 |
| CROATIA-Split | 496 | 42.9 | 45.0 (14.7) | 18-85 |  | 979 (404) | 94-2831 | 0730-0900 |
| CROATIA-Vis | 892 | 43.5 | 56.4 (15.5) | 18-93 |  | 622 (230) | 64-1820 | 0730-0900 |
| Rotterdam Study | 6497 | 43.6 | 63.3 (9.6) | 45.5-106 |  | 359 (115) | 14-966 | 0800-1100 |
| HBCS1934-44 | 451 | 36.1 | 60.61 (2.80) | 56 - 67 |  | 393 (120) | 125-990 | 0750-1055 |
| NFBC1966 | 1324 | 0 | 31.1 (0.3) | 30.3-32.4 |  | 380 (160) | 40-2370 | 0800-1100 |
| ALSPAC | 1,567 | 50.3 | 15.43 (0.26) | 14-17 |  | 486 (174) | 58-1683 | 0800-1057 |
| PIVUS | 919 | 50.2 | 70.2 (0.17) | 69-72 |  | 386 (125) | 31-930 | 0800-1000 |
| PREVEND | 1151 | 50.6 | 49.4 (13.0) | 28-75 |  | 442 (201) | 20-1734 | 0800-1100 |
| ET2DS | 1048 | 51.3 | 67.9 (4.2) | 60-74 |  | 731 (190) | 102-1447 | 0800-0830 |
| Raine Study | 860 | 51.9 | 17.1 (0.29) | 16-18 |  | 614 (235) | 36-1654 | Awakening (before 1000) |
| MrOS Sweden | 969 | 100 | 75.3 (3.2) | 70-81 |  | 487 (133) | 70-1550 | 0700-1000 |
| VIKING | 2073 | 39.9 | 49.9 (15.2) | 18-91 |  | 292 (170) | 7-1885 | 0800-1030 |
| SHIP | 910 | 44.7 | 49.8 (13.8) | 20-81 |  | * | * | Before 1300 |
| TwinsUK | 5654 | 0 | 53.3 (13.8) | 18-84 |  | * | * | 0800-1200 |
| KORA | 1651 | 48.6 | 60.92 (8.7) | 31-77 |  | * | * | N/A |

**Table S2. Genotyping methods used in each cohort**

| Cohort | Genotyping platform | Calling Algorithm | Reference Panel |
| --- | --- | --- | --- |
| ORCADES | Illumina HumanHap300 | MACH | Haplotype Reference Consortium |
| CROATIA-Korcula | Illumina HumanCNV370 | MACH | 1000 Genomes Phase 3 |
| CROATIA-Split | Illumina HumanCNV370 | MACH | 1000 Genomes Phase 3 |
| CROATIA-Vis | Illumina HumanHap300 | MACH | 1000 Genomes Phase 3 |
| Rotterdam Study | Illumina HumanHap 550v3 and Illumina HumanHap 610 | MACH | 1000 Genomes Phase 3 |
| HBCS1934-44 | Modified Illumina 610k | MACH | 1000 Genomes Phase 3 |
| NFBC1966 | Illumina HumanCNV370DUO | IMPUTE | 1000 Genomes Phase 3 |
| ALSPAC | Illumina HumanHap550K | MACH | 1000 Genomes Phase 3 |
| PIVUS | merged Human Omni Express and MetaboChip | IMPUTE2 | 1000 Genomes Phase 3 |
| PREVEND | Illumina Cyto SNP12 v2 array | Beagle 3.3.1 | 1000 Genomes Phase 3 |
| ET2DS | Illumina HumanHap300 | MACH | 1000 Genomes Phase 3 |
| Raine Study | Illumina Human660W-Quad | MACH | 1000 Genomes Phase 3 |
| MrOS Sweden | Illumina HumanOmni1_Quad_v1-0 B array | IMPUTE2 | 1000 Genomes Phase 3 |
| VIKING | Illumina HumanOmniExpressExome8v1-2_A | MACH | 1000 Genomes Phase 3 |
| SHIP | Affymetrix 6.0 | Impute | 1000 Genomes Phase 3 |
| TwinsUK | Illumina HumanHap300 | MACH | 1000 Genomes Phase 3 |
| KORA | Affymetrix 6.0 | Impute | 1000 Genomes Phase 3 |

**Table S3. Details of the disease and traits in UK Biobank investigated in the bidirectional two sample Mendelian randomization analyses**

| Exposure | Cases | Controls | Sample size | Number of SNPs used in instrument |
| --- | --- | --- | --- | --- |
| Osteoporosis | 5266 | 331893 | 337159 | 8 |
| Body mass index |  |  | 336107 | 287 |
| Diabetes mellitus | 16183 | 320290 | 336473 | 44 |
| Myocardial infarction | 7790 | 328893 | 336683 | 11 |
| Chronic ischaemic heart disease | 8755 | 328444 | 337199 | 15 |

**Table S4. Partitioned heritability**

| Cell Type | Prop._SNPs | Prop._h2 | Prop._h2_std_error | Enrichment | Enrichment_std_error | Enrichment_p |
| --- | --- | --- | --- | --- | --- | --- |
| Adrenal_Pancreas | 0.09 | 1.63 | 2.82 | 17.43 | 30.14 | 0.59 |
| Cardiovascular | 0.11 | 0.55 | 1.78 | 4.95 | 16.05 | 0.81 |
| CNS | 0.15 | 0.84 | 2.02 | 5.63 | 13.56 | 0.73 |
| Connective_Bone | 0.11 | 0.86 | 1.94 | 7.52 | 16.87 | 0.70 |
| GI | 0.17 | 2.35 | 3.74 | 14.01 | 22.32 | 0.56 |
| Immune | 0.23 | -0.14 | 1.84 | -0.61 | 7.87 | 0.84 |
| Kidney | 0.04 | 1.92 | 2.88 | 45.03 | 67.65 | 0.52 |
| Liver | 0.07 | 2.77 | 4.63 | 38.38 | 64.17 | 0.56 |
| Other | 0.20 | -0.01 | 2.10 | -0.05 | 10.35 | 0.92 |
| SkeletalMuscle | 0.10 | -0.05 | 1.62 | -0.45 | 15.60 | 0.93 |

**Table S5. Pathway-based association analysis**

| Pathway name | P value |
| --- | --- |
| REACTOME_METABOLISM_OF_LIPIDS_AND_LIPOPROTEINS | 1.35E-05 |
| REACTOME_SPHINGOLIPID_METABOLISM | 1.65E-04 |
| KEGG_ALPHA_LINOLENIC_ACID_METABOLISM | 2.61E-04 |
| BIOCARTA_EGFR_SMRTE_PATHWAY | 3.65E-04 |
| REACTOME_PHOSPHOLIPID_METABOLISM | 5.13E-04 |
| REACTOME_VOLTAGE_GATED_POTASSIUM_CHANNELS | 6.73E-04 |
| REACTOME_GLYCOSPHINGOLIPID_METABOLISM | 1.42E-03 |
| KEGG_TGF_BETA_SIGNALING_PATHWAY | 1.73E-03 |
| REACTOME_BETA_DEFENSINS | 2.41E-03 |
| KEGG_STARCH_AND_SUCROSE_METABOLISM | 2.51E-03 |
| REACTOME_CIRCADIAN_CLOCK | 2.73E-03 |
| REACTOME_BMAL1_CLOCK_NPAS2_ACTIVATES_CIRCADIAN_EXPRESSION | 3.21E-03 |
| REACTOME_DEFENSINS | 3.64E-03 |
| REACTOME_CIRCADIAN_REPRESSION_OF_EXPRESSION_BY_REV_ERBA | 4.60E-03 |
| KEGG_SPHINGOLIPID_METABOLISM | 4.66E-03 |

**Table S6. Cis-eQTLs (q-value ≤ 0.05) identified for *SERPINA6* that were at genome wide significance in CORNET (p-value ≤ 5x10^-8^)**

| SNP | Genomic Loci | Kruskal Wallis | p-value | q-value | CORNET p-value |
| --- | --- | --- | --- | --- | --- |
| rs2736898 | 14:94823817 | 40.68215 | 1.47E-09 | 0.000153 | 7.03E-14 |
| rs3762132 | 14:94834575 | 39.24606 | 3.00E-09 | 0.000305 | 1.57E-13 |
| rs59036614 | 14:94830448 | 38.45662 | 4.46E-09 | 0.000441 | 9.49E-14 |
| rs2749529 | 14:94820459 | 38.41395 | 4.56E-09 | 0.000445 | 9.92E-14 |
| rs2749527 | 14:94827068 | 38.34012 | 4.73E-09 | 0.000445 | 1.75E-13 |
| rs2013150 | 14:94825769 | 38.34473 | 4.72E-09 | 0.000445 | 7.12E-14 |
| rs941594 | 14:94835914 | 38.04932 | 5.47E-09 | 0.000508 | 1.45E-13 |
| rs2736899 | 14:94823220 | 37.93856 | 5.78E-09 | 0.000522 | 9.51E-14 |
| rs2749530 | 14:94816299 | 37.74902 | 6.35E-09 | 0.000551 | 1.40E-13 |
| rs1243171 | 14:94836784 | 37.44135 | 7.41E-09 | 0.000635 | 2.02E-13 |
| rs1243173 | 14:94836298 | 36.55909 | 1.15E-08 | 0.000947 | 1.53E-13 |
| rs2749539 | 14:94803365 | 28.66661 | 5.96E-07 | 0.034184 | 3.04E-08 |
| rs4491436 | 14:94804700 | 28.2968 | 7.17E-07 | 0.0381 | 5.97E-19 |
| rs718187 | 14:94801860 | 28.2968 | 7.17E-07 | 0.0381 | 4.52E-19 |
| rs9989237 | 14:94795202 | 28.2968 | 7.17E-07 | 0.0381 | 2.16E-19 |
| rs12589136 | 14:94793686 | 28.2968 | 7.17E-07 | 0.0381 | 3.23E-19 |
| rs6575415 | 14:94791601 | 28.2968 | 7.17E-07 | 0.0381 | 2.97E-19 |
| rs2281518 | 14:94789117 | 28.2968 | 7.17E-07 | 0.0381 | 4.58E-19 |
| rs941599 | 14:94788341 | 28.2968 | 7.17E-07 | 0.0381 | 4.41E-19 |
| rs4905187 | 14:94805193 | 28.2968 | 7.17E-07 | 0.0381 | 7.34E-19 |
| rs7161521 | 14:94787288 | 28.2968 | 7.17E-07 | 0.0381 | 3.07E-19 |

**Table S7: Colocalisation of GWAMA and *SERPINA6* cis-eQTL signal using Coloc**

| LD_block | nsnps | PP.H0.abf | PP.H1.abf | PP.H2.abf | PP.H3.abf | PP.H4.abf |
| --- | --- | --- | --- | --- | --- | --- |
| LD1 | 4 | 4.16E-05 | 7.82E-07 | 0.259387 | 0.004138 | 0.736433 |
| LD2 | 13 | 4.81E-14 | 1.55E-10 | 2.92E-06 | 0.008404 | 0.991593 |
| LD3 | 2 | 5.14E-05 | 1.34E-06 | 0.047832 | 0.000298 | 0.951817 |
| LD4 | 52 | 2.24E-17 | 5.87E-16 | 0.00038 | 0.008972 | 0.990648 |
| All_SNPs | 535 | 9.76E-18 | 3.48E-14 | 0.000167 | 0.594 | 0.406 |

**Table** **S7:** Analysis using Coloc, a Bayesian test for colocalisation. Coloc Approximate Bayes Factor Colocalisation Analysis (ABF) return 5 hypothesis tests to determine if two genetic association signals share the same causal variant. H0.abf: neither trait has a genetic association in the region, H1.abf: only cis-eQTL has a genetic association in the region. H2.abf: only GWAMA has a genetic association in the region, H3: both traits are associated, but with different causal variants. H4: both traits are associated and share a single causal variant. This test assumes a single shared causal variant for both signals. Posterior probability (PP) of a shared causal variant is low when examining all SNPs together (40.6%) but increases in certain LD blocks when examined individually. Strongest signal is present in LD blocks 2 (99.2%) and 4 (99.1%).


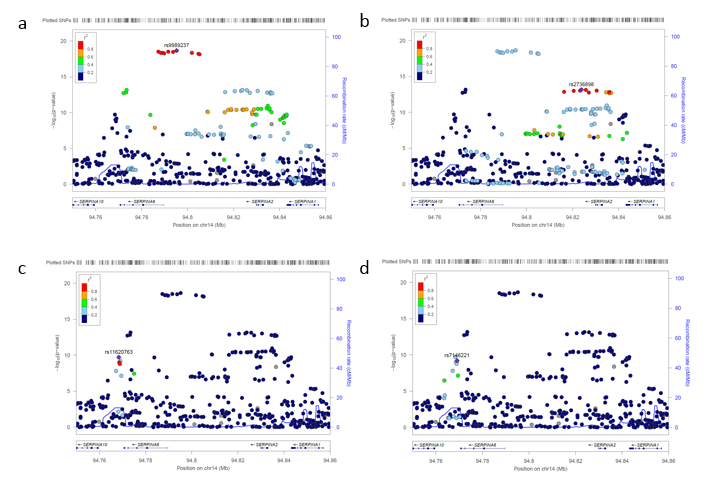


**Figure S1.** Zoomed in Manhattan plot (LocusZoom plot) of -log10 P values of the SNP-based association analysis of morning plasma cortisol (n=25,314). The four plots show the four LD blocks (r^2^ > 0.3) in this locus with the top SNP labelled in each block.

**Figure S2**. Genetic correlations calculated using linkage disequilibrium score regression between plasma cortisol in the CORNET cohort (n=25,314) and traits and diseases from UK Biobank. Whiskers are 95% CI. The *r_g_* (95% CI) for BMI -0.32 (-0.54 to -0.10), diabetes mellitus -0.09 (-0.30 to 0.12), osteoporosis 0.05 (-0.32 to 0.41), chronic ischaemic heart disease 0.30 (-0.02 to 0.62) and myocardial infarction 0.50 (0.04 to 0.97).


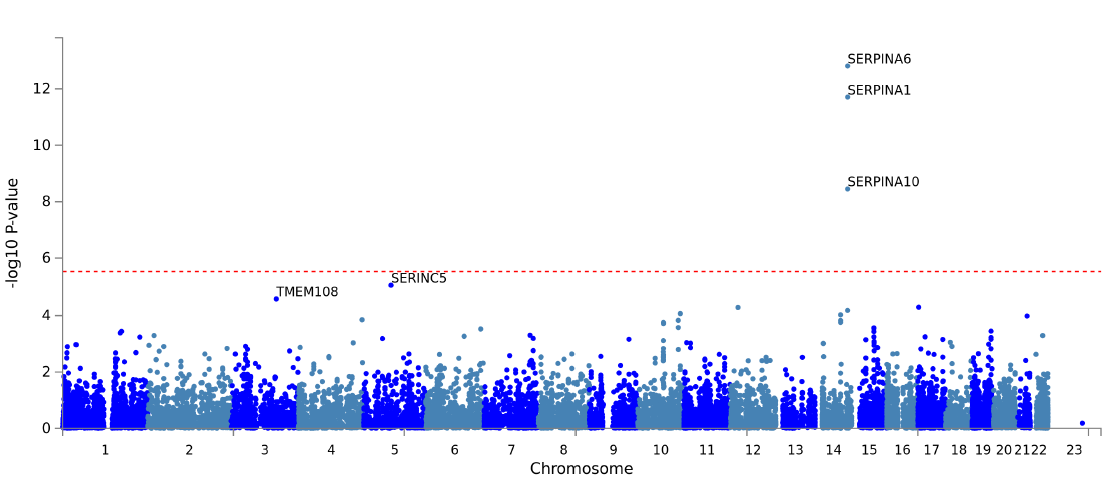


**Figure S3.** Manhattan plot of the gene-based association analysis. Three genes *SERPINA6* (p=1.07 x 10^-10^), *SERPINA1* (p=2.20 x 10^-10^), *SERPINA1* (p=4.91 x 10^-7^), all located on chromosome 14, that attained genome-wide significance following correction for multiple comparisons.


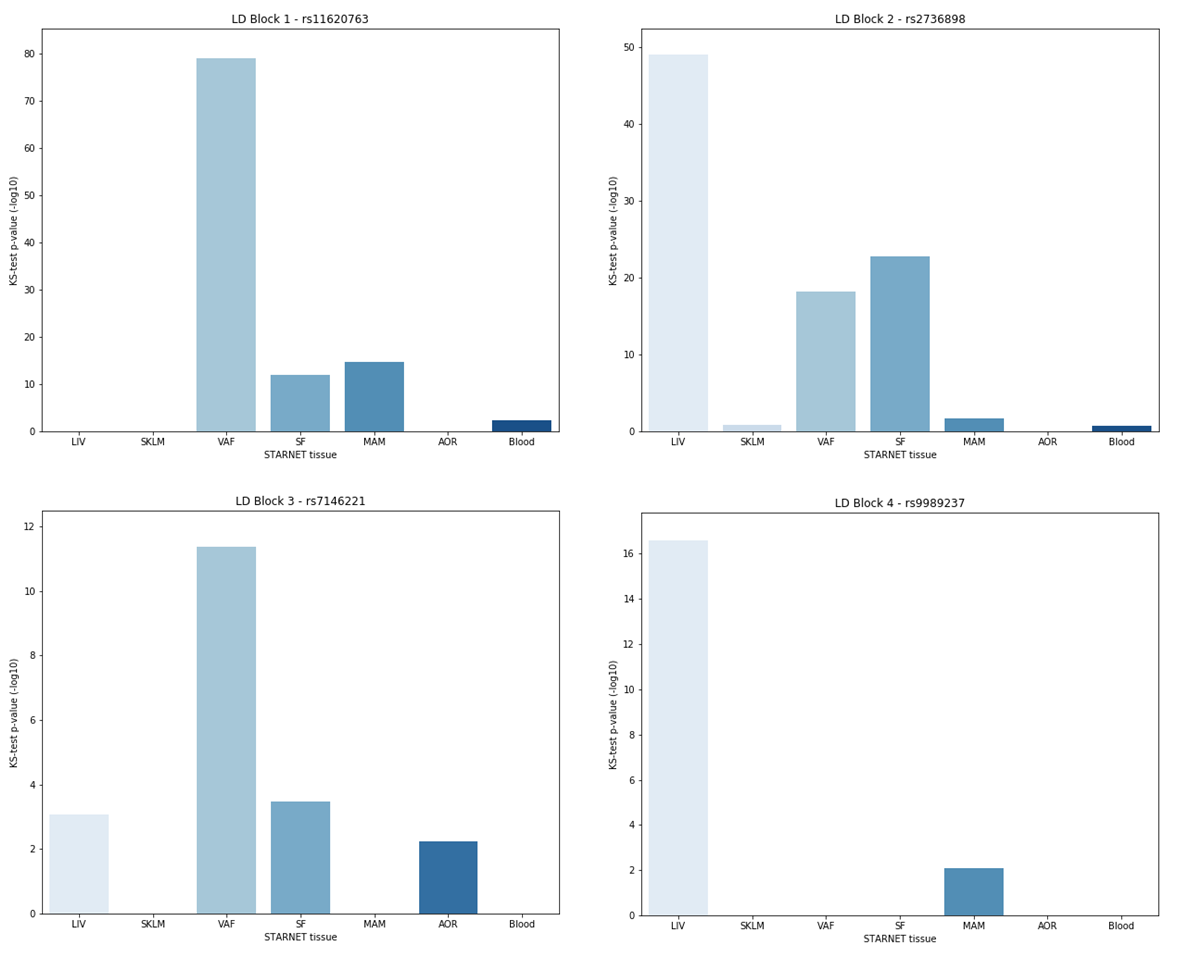


**Figure S4.** Kolmogorov-Smirnov test for *SERPINA6* expression of the lead SNP in each LD block in multiple tissues


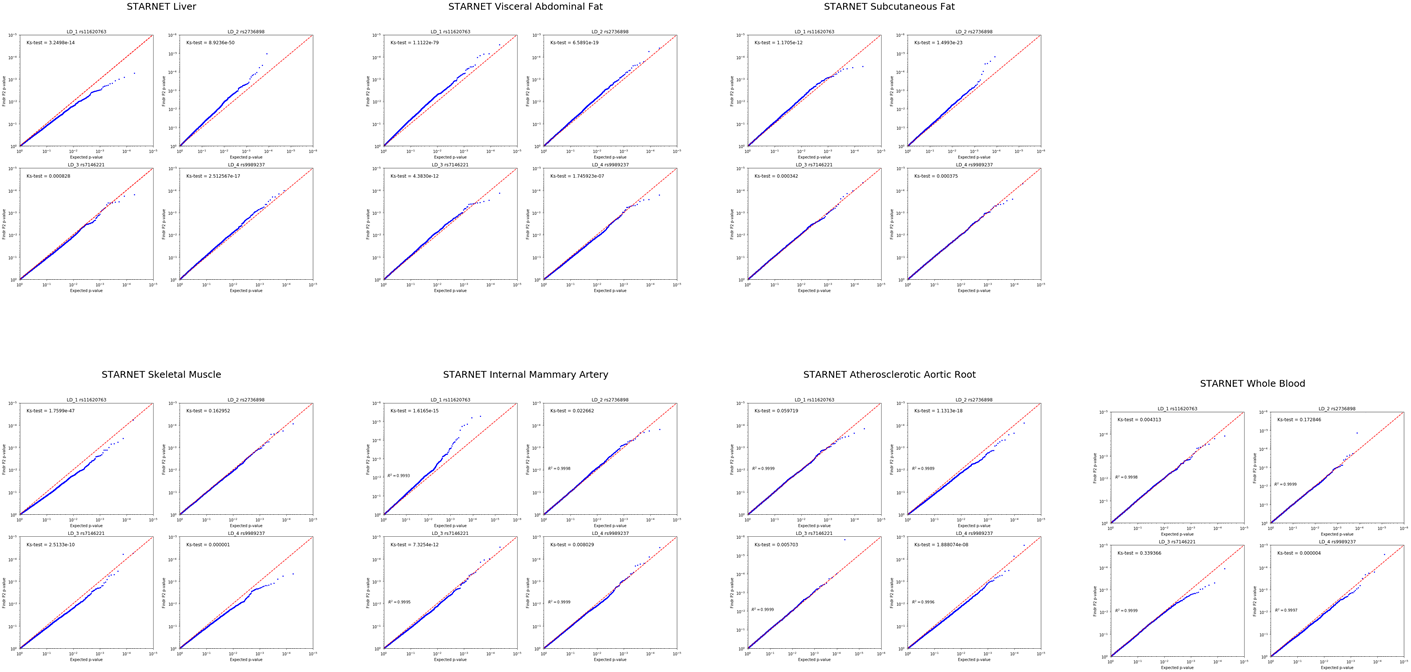


**Figure S5.** Quantile-quantile plot of tissue-specific *SERPINA6* expression according to each LD block


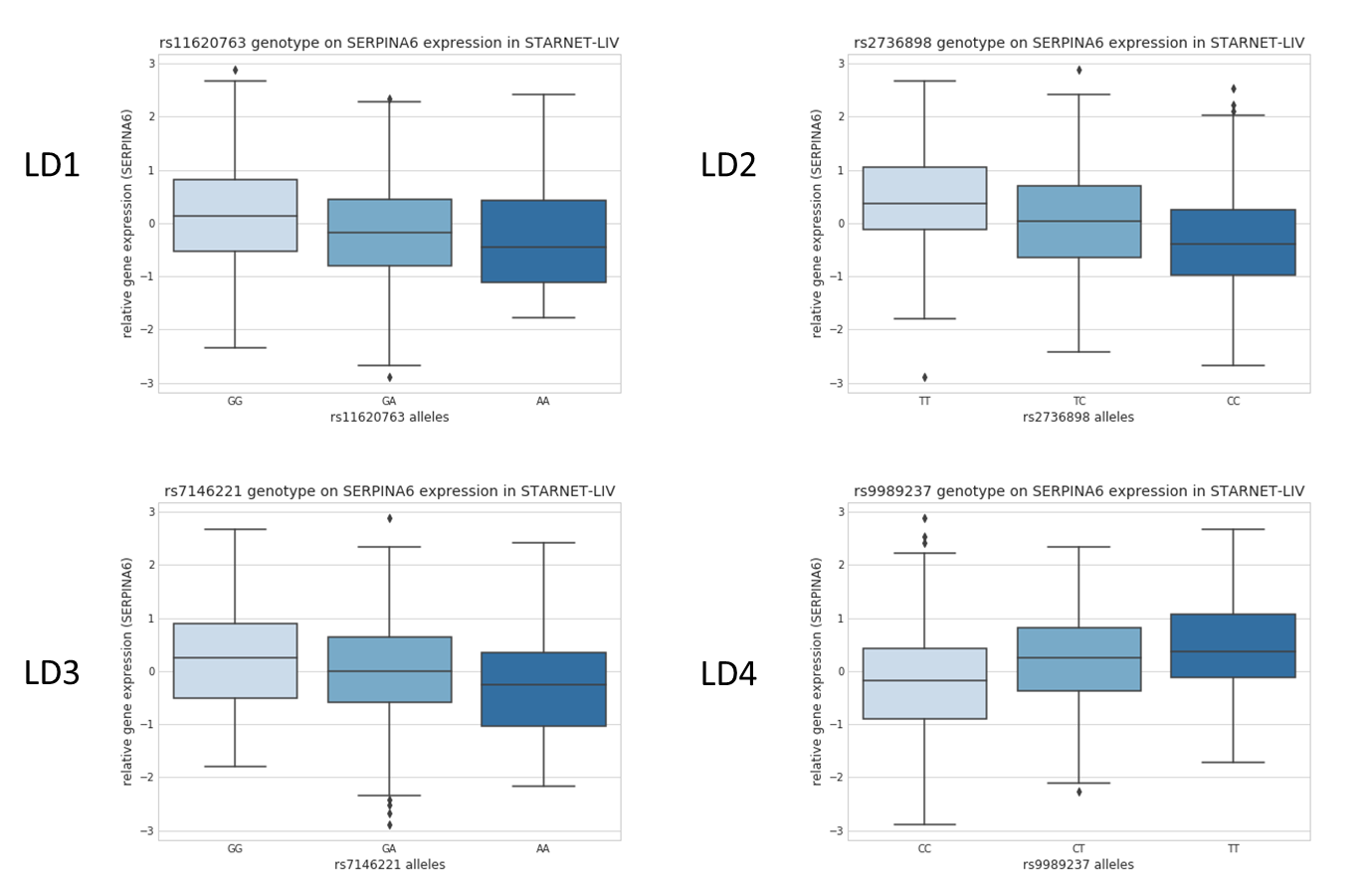


**Figure S6.** Magnitude and allele-specific effects of lead SNP in each LD block on *SERPINA6* expression in liver


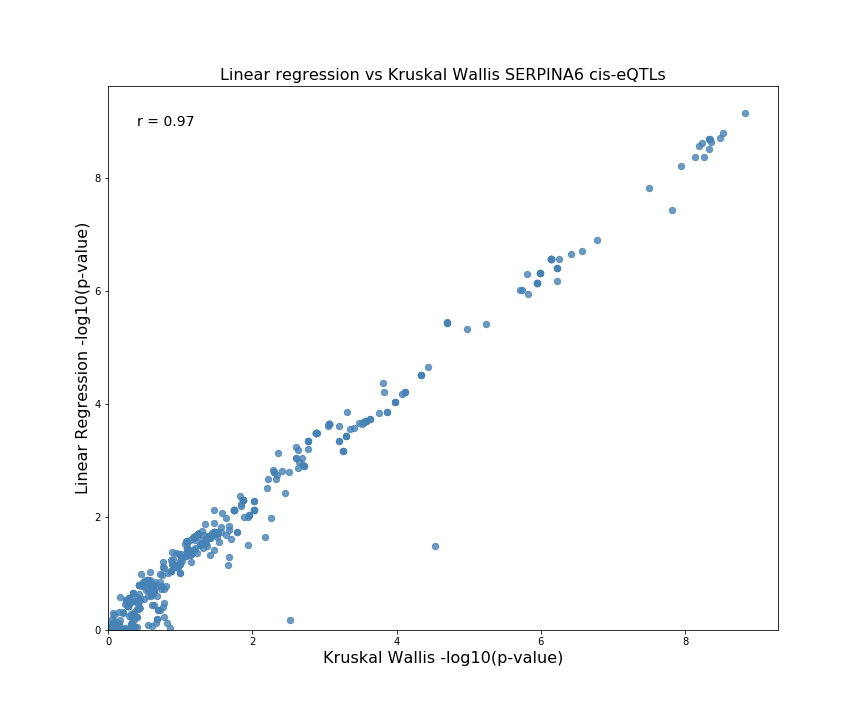


**Figure S7.** Comparison of -log_10_ p-values for *SERPINA6* cis-eQTLs calculated using Linear regression vs Kruskal Wallis. Spearman’s R = 0.97.

References

1. Hofman A, van Duijn CM, Franco OH, Ikram MA, Janssen HLA, Klaver CCW, et al. The Rotterdam Study: 2012 objectives and design update. Eur J Epidemiol. 2011;26: 657. doi:10.1007/s10654-011-9610-5

2. Rantakallio P. Groups at risk in low birth weight infants and perinatal mortality. Acta paediatrica Scandinavica. 1969;193: Suppl 193:1+.

3. Boyd A, Golding J, Macleod J, Lawlor DA, Fraser A, Henderson J, et al. Cohort Profile: The ‘Children of the 90s’—the index offspring of the Avon Longitudinal Study of Parents and Children. Int J Epidemiol. 2013;42: 111–127. doi:10.1093/ije/dys064

4. Price JF, Reynolds RM, Mitchell RJ, Williamson RM, Fowkes FGR, Deary IJ, et al. The Edinburgh Type 2 Diabetes Study: study protocol. BMC Endocrine Disorders. 2008;8: 18. doi:10.1186/1472-6823-8-18

5. Finucane HK, Bulik-Sullivan B, Gusev A, Trynka G, Reshef Y, Loh P-R, et al. Partitioning heritability by functional annotation using genome-wide association summary statistics. Nature Genetics. 2015;47: 1228–1235. doi:10.1038/ng.3404

6. Haycock PC, Burgess S, Wade KH, Bowden J, Relton C, Davey Smith G. Best (but oft-forgotten) practices: the design, analysis, and interpretation of Mendelian randomization studies. Am J Clin Nutr. 2016;103: 965–978. doi:10.3945/ajcn.115.118216

7. Hartwig FP, Davies NM, Hemani G, Davey Smith G. Two-sample Mendelian randomization: avoiding the downsides of a powerful, widely applicable but potentially fallible technique. Int J Epidemiol. 2016;45: 1717–1726. doi:10.1093/ije/dyx028

8. Bowden J, Smith GD, Haycock PC, Burgess S. Consistent Estimation in Mendelian Randomization with Some Invalid Instruments Using a Weighted Median Estimator. Genetic Epidemiology. 2016;40: 304–314. doi:10.1002/gepi.21965

9. Bowden J, Davey Smith G, Burgess S. Mendelian randomization with invalid instruments: effect estimation and bias detection through Egger regression. Int J Epidemiol. 2015;44: 512–525. doi:10.1093/ije/dyv080
